# Stable training via elastic adaptive deep reinforcement learning for autonomous navigation of intelligent vehicles

**DOI:** 10.1038/s44172-024-00182-8

**Published:** 2024-02-26

**Authors:** Yujiao Zhao, Yong Ma, Guibing Zhu, Songlin Hu, Xinping Yan

**Affiliations:** 1 State Key Laboratory of Maritime Technology and Safety, Wuhan University of Technology, Wuhan, China; 2 School of Navigation, Wuhan University of Technology, Wuhan, China; 3 National Engineering Research Center for Water Transport Safety, Wuhan University of Technology, Wuhan, China; 4 Chongqing Research Institute, Wuhan University of Technology, Chongqing, China; 5 Sanya Science and Education Innovation Park, Wuhan University of Technology, Sanya, China; 6 Marine College, Zhejiang Ocean University, Zhoushan, China; 7 Institute of Advanced Technology, Nanjing University of Posts and Telecommunications, Nanjing, China; 8 Intelligent Transportation Systems Research Center, Wuhan University of Technology, Wuhan, China

**Keywords:** Engineering, Mathematics and computing

## Abstract

The uncertain stability of deep reinforcement learning training on complex tasks impedes its development and deployment, especially in intelligent vehicles, such as intelligent surface vessels and self-driving cars. Complex and varied environmental states puzzle training of decision-making networks. Here we propose an elastic adaptive deep reinforcement learning algorithm to address these challenges and achieve autonomous navigation in intelligent vehicles. Our method trains the decision-making network over the function and optimization learning stages, in which the state space and action space of autonomous navigation tasks are pruned by choosing classic states and actions to reduce data similarity, facilitating more stable training. We introduce a task-adaptive observed behaviour classification technique in the function learning stage to divide state and action spaces into subspaces and identify classic states and actions. In which the classic states and actions are accumulated as the training dataset that enhances its training efficiency. In the subsequent optimization learning stage, the decision-making network is refined through meticulous exploration and accumulation of datasets. The proposed elastic adaptive deep reinforcement learning enables the decision-making network to effectively learn from complex state and action spaces, leading to more efficient training compared to traditional deep reinforcement learning approaches. Simulation results demonstrate the remarkable effectiveness of our method in training decision-making networks for intelligent vehicles. The findings validate that our method provides reliable and efficient training for decision-making networks in intelligent vehicles. Moreover, our method exhibits stability in training other tasks characterized by continuous state and action spaces.

## Introduction

The autonomous navigation techniques of intelligent surface vessels and self-driving cars are epoch-making achievements, which is a key link in achieving inexpensive unmanned transportation^1^. It aims to improve operation efficiency, strengthen operation safety, and liberate manpower through the abilities of perception and independent decision-making^2–5^. Autonomous navigation is the premise of realizing the intelligent surface vessel and self-driving car. However, achieving high-quality autonomous navigation requires abundant computational power and time costs, which hinders the rapid iteration of intelligent surface vessels and self-driving cars technologies. Moreover, restricted by the limitation of human experience and the difficulty of massive experience data acquisition, the existing autonomous navigation technologies are far from the human level. The poor efficiency of the navigation system, the inadaptability to complex navigation situations and unknown situations, and the instability of controller switching have not been effectively resolved, which impediments the practical application of autonomous driving technology. Due to the DRL-based approaches being limited by computational power and training samples, traditional control approaches still dominate in autonomous driving technology^6–8^. Yet, deep reinforcement learning (DRL)-based approaches have great potential by their abilities of learning and end-to-end feature extraction^9^.

Although DRL performs satisfactorily in various tasks, there are still problems in its application to autonomous driving of intelligent surface vessels and self-driving cars, such as hardly explored environment states^10^, verbose obstacle information, unprecise collision risk assessment, and reward functions with negative internal interaction^11,12^. Moreover, due to the complexity of environments and tasks^13^, the commonly used exploration strategies such as *ϵ* − *greedy* algorithm and Ornstein Unlenbeck stochastic process are inefficient and useless to explore the navigation environment. Furthermore, for the collision risk assessment, the distance at the closest point of approach and the time to the closest point of approach are difficult to accurately quantify and compare the collision risks of vessels in multi-vessel encounter situations, although they are the most popular assessment indicators of the vessel collision risk^14^. Furthermore, changes in obstacle situations can result in sudden fluctuations in input values and control gains for the control system. These fluctuations can have a serious impact on the stability and operational efficiency of the control system, potentially leading to accidents or crashes. Therefore, it is attractive and valuable to achieve autonomous navigation of intelligent surface vessels.

Autonomous navigation of intelligent vehicles is confronted with enormous challenges. Taking an intelligent surface vessel as an example, the path following^15,16^ can sometimes hinder the vessel from effectively performing collision avoidance maneuvers in complex situations. Nevertheless, the complex environment and situations are hard to explore, predict, and quantify^17–19^, which affects the decision-making efficiency and success rate of vessels in multi-vessel encounter scenarios. More importantly, the training of decision-making networks cannot guarantee stability due to changes in environment states. The reward value can reflect the training effect of the decision-making network in a certain^20,21^. It can be found that reward values are prone to fluctuations during training in some complex tasks, which reflects the instability of decision-making network training. Although extensive navigation practices have accumulated rich collision avoidance experiences and theories regarding vessel collision avoidance^22,23^, there is a difficulty to get the ability for machines, especially in the coexisting scenarios of intelligent surface vessels and manned surface vessels. Therefore, it is particularly meaningful to train intelligent surface vessels to follow the Convention on the International Regulations for Preventing Collisions at Sea (COLREGs). The existing collision avoidance methods, including artificial potential field (APF), dynamic path planning^24,25^, and communication negotiation, are restricted by efficiency, equipment and computation cost, which are incapable of making optimal collision avoidance decisions in time. Solutions based on machine learning with prominent behavior learning and feature extraction ability are becoming new hot technologies in various fields^7,26,27^. Among them, DRL has been the most potential solution for intelligent surface vessel autonomous navigation^28–30^, which can autonomously and independently learn and make decisions in complex environments without communication^31–33^.

**Fig. 1:**
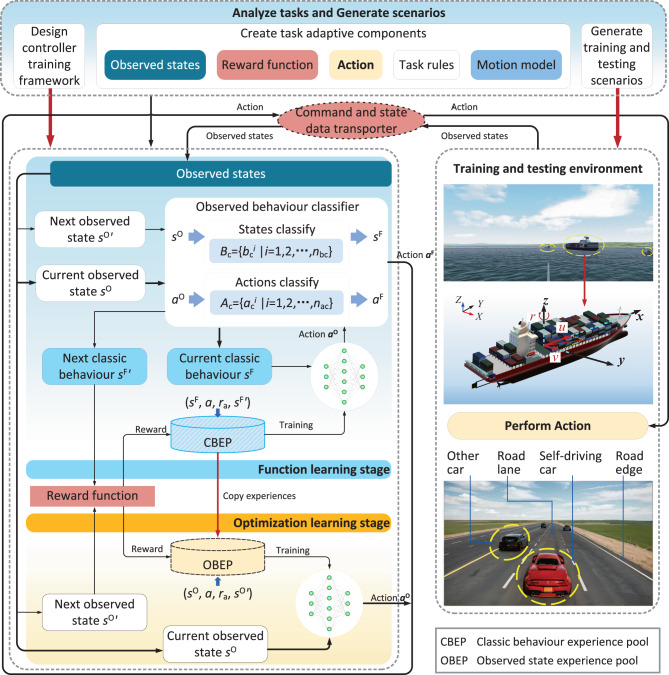
Overview of elastic adaptive deep reinforcement learning. *B*_c_ and *A*_c_ represent the sets of classic states and classic actions, respectively. *n*_bc_ and *n*_ac_ denote the number of classic states and classic actions, respectively. *s*^F^ and *s*^O^ are the input states of the decision-making network in the function and optimization learning stages, respectively. *a* is the performed action, *r*_a_ is the reward. ${s}^{{{{{{{{\rm{F}}}}}}}}{\prime} }$ and ${s}^{{{{{{{{\rm{O}}}}}}}}{\prime} }$ are the input states after performing action. *a*^F^ and *a*^O^ are the output actions of the decision-making network in the function and optimization learning stages, respectively. *u*, *v*, *r* are the surge velocity, sway velocity, and yaw angle rate of the intelligent surface vessel.

In this Article, we address the autonomous navigation of intelligent vehicles by an elastic adaptive deep reinforcement learning (EADRL) with consideration of the versatility and practicability of the decision-making algorithm. The essential idea of the proposed EADRL lies in its unique methodology for streamlining the complexity of environmental exploration. This is achieved through a strategic partitioning of state/action spaces based on observed behavior classifications. The key innovation of EADRL is to replace a large number of non-classic behaviors with a small amount of classic behaviors fuzziness, in order to achieve pruning of the state space and action space. This differentiation allows for a more efficient and targeted learning process, where neural networks are trained predominantly with classic behavior data, recognized for their relevance and utility in decision-making scenarios. The architecture of EADRL is bifurcated into two distinct stages: the function learning stage and the optimization learning stage (Fig. 1). At the function learning stage, the technique of observed behavior classification plays a pivotal role. It not only helps in segregating the data into more manageable and relevant subsets but also reduces the order of magnitude of environment states need to be explored. A meaningful advantage of EADRL is its efficient and stable exploration to notably reduce the number of states needed to be explored and the uncertainty of exploration experiences, surpassing traditional DRL methods. (The uncertain stability and complex tasks in the navigation of autonomous vehicles are discussed in Supplementary Note 1.) Our approach can be applied to complex navigation environments of different type of vehicles, including multiple intelligent surface vessels encountering, intelligent surface vessels path following and self-driving cars passing multiple highways and intersections, which cannot be achieved by previous scenario-based approaches. The structure of EADRL is summarized in Fig. 1.

## Results

To demonstrate the effectiveness of the proposed EADRL approach, we trained individual decision-making networks for intelligent surface vessels and self-driving cars with large-scale naturalistic navigation datasets and conducted simulation experiments. For the sake of explanation, we will use intelligent surface vessels with greater difficulty in control as the research object to explain EADRL in the context. The self-driving car is introduced as an extended application scenario.

### EADRL guarantees intelligent surface vessels to navigate in a multi-vessel encounter situation

Intelligent surface vessels autonomously navigate in a realistic multi-vessel encounter situation that happened in early March 2022 near the Eastern Boarding Ground of Singapore. The nearest encounter distance was only 1.3 times that of the length of the vessel, which had seriously violated the safe domain of other vessels and threatened navigation safety.

**Fig. 2:**
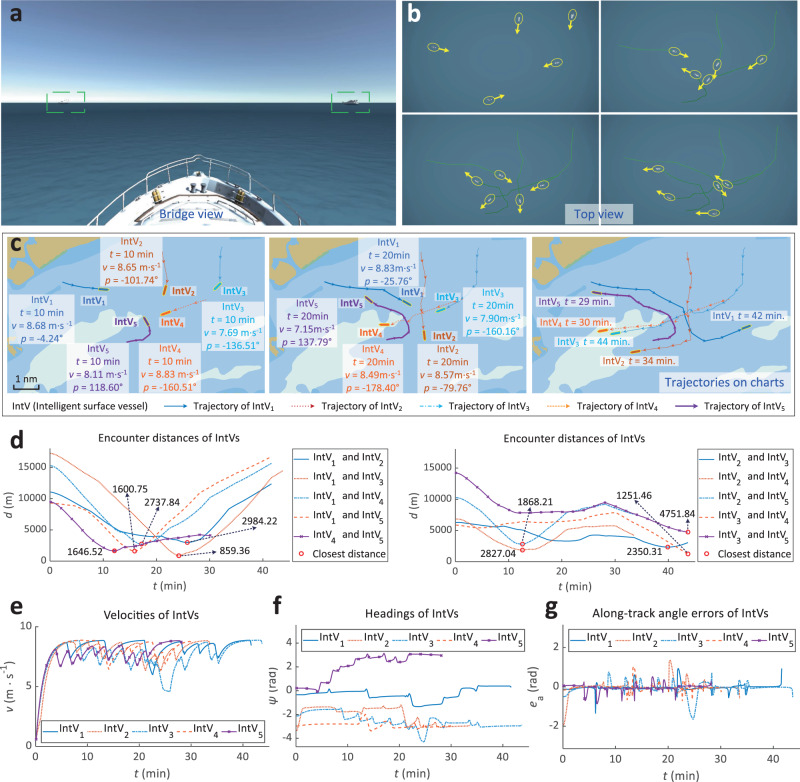
Qualitative results of elastic adaptive deep reinforcement learning in intelligent surface vessel scenario. **a** Bridge view of intelligent surface vessel in simulation. **b** Top view of intelligent surface vessels. The evolution of the encounter situation of five intelligent surface vessels. **c** Trajectories of intelligent surface vessels and encounter situations at different moments. “nm" means nautical mile. **d** Encounter distances of intelligent surface vessels. Encounter distances between every two intelligent surface vessels are safe enough. Velocities of intelligent surface vessels are maintained in [6.4, 8.8] m ⋅ s^−1^. **e** Velocities of intelligent surface vessels. The lowest velocity appears during the collision avoidance process between IntV_1_ and IntV_3_. **f** Headings of intelligent surface vessels. Headings of intelligent surface vessels are maintained steady. **g** Along-track angle errors of intelligent surface vessels. Along-track angle errors of intelligent surface vessels fluctuate when turning for collision avoidance and near path points and maintain steady at other times.

The intelligent surface vessel autonomous navigation environment is exceptionally complicated in the multi-vessel encounter scenario. Figure 2a, b show the bridge view and top view of intelligent surface vessels autonomous navigation in a virtual-real environment, respectively. As illustrated in Fig. 2c, five intelligent surface vessels formed different encounter situations constantly. At *t* = 1 min, five intelligent surface vessels navigated on their preset paths with collision-free. At *t* = 10 min, five intelligent surface vessels gradually approached each other, IntV_4_ and IntV_5_ appeared on the preset paths of IntV_2_ and IntV_4_, respectively. Figure 2d shows that encounter distances were safe enough for the intelligent surface vessels. Therefore, IntV_2_ and IntV_3_ did not change their navigation states. At *t* = 10 min, IntV_2_ detected IntV_4_ and took a deceleration action, and then IntV_2_ was out of the encounter situation with IntV_4_ at *t* = 13 min. At *t* = 12 min, IntV_4_ detected IntV_5_, and IntV_4_ decelerated to give way to IntV_5_. IntV_1_ and IntV_3_ formed a new crossing encounter situation at *t* = 20 min. IntV_3_ took actions to pass around the stern of IntV_1_. Figure 2e–g present the velocities, headings, and along-track angle errors of intelligent surface vessels, respectively. In the whole process of collision avoidance, intelligent surface vessels navigated at the velocity of up to 17 knots, and the lowest velocity was 8.9 knots. The nearest encounter distance between the five intelligent surface vessels was 859.3557 m, generated by IntV_1_ and IntV_3_ at *t* = 24 min. (More detailed verification results are shown in Supplementary Note 3.).

### EADRL drives car on complex roads

**Fig. 3:**
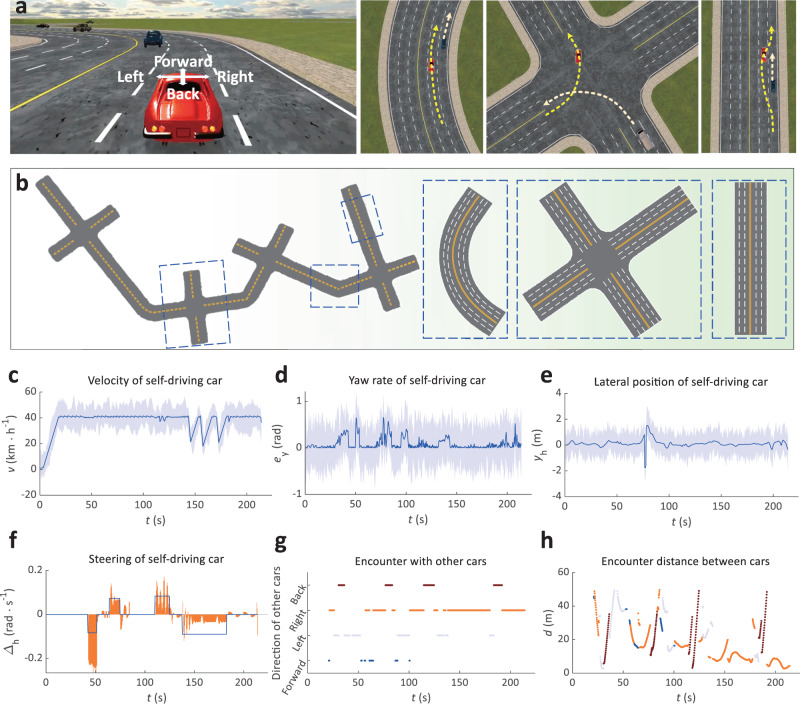
Qualitative results of elastic adaptive deep reinforcement learning in self-driving car scenario. **a** Front view and top-down view of self-driving car on road. The self-driving car is driven in a test environment where other cars exist. **b** Test road of a self-driving car. The test road consists of straight, curved, and intersection roads. **c** Velocities of self-driving car. **d** Steerings of a self-driving car. **e** Yaw rates of self-driving car. The light gray shaded regions in **c–e** represent the 85% confidence level. **f** Lateral positions of self-driving car. **g** Relative orientation between self-driving car and other cars. **h** Encounter distance between self-driving car and other cars.

Figure 3 shows the results of the three-lane highway environment for the EADRL. As shown in Fig. 3a, there are interferences from other cars on the test road which consists of straight lanes, bends, and intersections. Figure 3b shows the test road. Figure 3c shows that the velocity of the self-driving car is smoothly transforming, except that the velocity slows down during turns and encounters. As shown in Fig. 3d, the self-driving car steers its wheels following the lane turning. Figure 3e, f show the yaw rate and off-center distance in a lateral position, respectively. The yaw rate and off-center distance are fluctuated when the self-driving car turns near intersections. Figure 3g, h show the encounter situation between the self-driving car and other cars (relative direction (g) and distance (h)). It can be observed that the self-driving car navigates autonomously in busy traffic environments.

### Result analysis

**Fig. 4:**
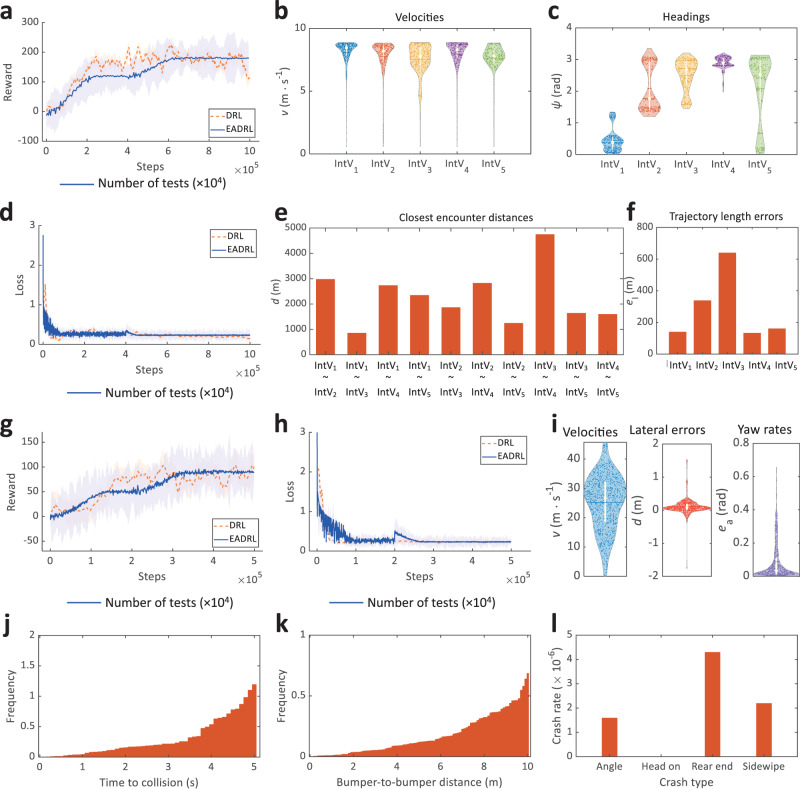
Quantitative results of elastic adaptive deep reinforcement learning in intelligent surface vessel and self-driving car test scenarios. **a** Rewards of decision-making agent training in intelligent surface vessel scenario. The light orange and light blue shaded regions in (**a**, **g**) represent the reward standard deviation of deep reinforcement learning and elastic adaptive deep reinforcement learning, respectively. **b V**elocities of intelligent surface vessels. **c** Heading angles of intelligent surface vessels. **d** Losses of decision-making agent training in intelligent surface vessel scenario. The light orange and light blue shaded regions in (**d**, **h**) represent the loss standard deviation of deep reinforcement learning and elastic adaptive deep reinforcement learning, respectively. **e** Encounter distances between intelligent surface vessels. **f** Trajectory length errors of intelligent surface vessels. **g** Rewards of decision-making agent training in self-driving car scenario. **h** Losses of decision-making agent training in self-driving car scenario. **i** Quantitative results of self-driving car. Numerical evaluation and various metrics (velocity, lateral error, and yaw rate) of self-driving cars. In the violin plots, the horizontal line mark indicates the median, and the bottom and top edges of the white box indicate the 25th and 75th percentiles, respectively. The white vertical line extends to the most extreme data points not considered outliers, and the outliers are plotted individually using the dots. The internal dots indicates the distribution density of data. **j** Time to collision. **k** Bumper-to-bumper distance. **l** Crash rate of each crash type.

Qualitative results of EADRL in intelligent surface vessel and self-driving car test scenarios are shown in Fig. 4. As shown in Fig. 4a, g, during the training process, the reward values gradually increase and converge to certain values. In contrast to the general DRL, reward values of the optimization stage of EADRL converge more stably, where the standard deviation of EADRL is 2.132, reduced than DRL 10.94 by ~80%. Worthy, compared to the dense deep reinforcement learning (D2RL)^1^, EADRL learns higher reward values faster in the function learning stage of training and exhibits more stable reward variations in the optimization learning stage (Details can be found in Supplementary Fig. 12). Figure 4d, h illustrate that the training loss of EADRL has two convergence processes, corresponding to the function learning stage and the optimization learning stage. Figure 4b, c, e, f show the velocities, headings, closest encounter distances, and trajectory length errors of intelligent surface vessels, respectively. It can be observed that EADRL navigates intelligent surface vessels safely. Figure 4i demonstrates that our approach can guarantee that self-driving car drives on highways safely. Moreover, we tested the performance of the EADRL to navigate self-driving cars under adverse conditions. Figure 4j–l confirm that EADRL can effectively ensure self-driving cars avoid collisions with other vehicles. For intelligent surface vessel scenarios, the trajectories comparison of intelligent surface vessels using EADRL and other high-performance algorithms are shown in Supplementary Figs. 2–8. APF can be used in collision avoidance rather than line-of-sight (LOS) or pure pursuit (PP). EADRL yields similar performance for velocity and heading maintenance to APF, but EADRL is prominently better than APF in encounter distance and trajectory length, where the trajectory length error of EADRL reduces 31% less than APF. Moreover, EADRL performs behaviors as conforming as possible to COLREGs, while APF is not.

## Discussion

Autonomous navigation plays a crucial role in the advancement and deployment of intelligent surface vessels and self-driving cars. While existing research has made notable contributions to realizing autonomous navigation, certain limitations persist. One prominent challenge in achieving autonomous navigation for intelligent surface vessels and self-driving cars is the abrupt changes in input and control gain resulting from dynamic alterations of obstacle scenarios. Such changes detrimentally impact controller performance, system stability, and can even lead to system failure. Moreover, the complex navigation environments encountered by intelligent surface vessels and self-driving cars introduce challenges related to the uncertainty of situational changes and the expansion of the state-space dimension. These factors pose serious obstacles for control theory-based approaches in accurately analyzing and predicting situational trends.

We have successfully demonstrated the applicability of EADRL in achieving autonomous navigation in multi-vessel encounter scenarios and self-driving car scenarios. Considering the stochastic and intricate nature of navigation environments, EADRL employs a staged approach to exploration and learning. By effectively categorizing observed behaviors, EADRL facilitates exploration and learning processes. Ultimately, EADRL enables the autonomous navigation of intelligent surface vessels and self-driving cars without the need for system or controller switching, thereby mitigating the risk of faults resulting from inaccurate situation assessment. Furthermore, even in multi-vessel encounter situations, EADRL can perform real-time collision risk assessment and adhere to COLREGs (International Regulations for Preventing Collisions at Sea) to execute deceleration and steering maneuvers, ensuring safe collision avoidance. This aspect provides a valuable reference for machine learning algorithms addressing surface vessel collision avoidance challenges. Distinguishing itself from conventional Deep Reinforcement Learning (DRL) methods, EADRL incorporates a two-stage approach that includes preliminary exploration and training in the function learning stage, followed by optimization learning employing DRL techniques. This staged methodology considerably streamlines the complexity of environmental exploration. EADRL initially classifies navigation behaviors to find a series of representative classic behaviors. By focusing on these representative classic behaviors, EADRL effectively reduces the uncertainty often associated with exploratory processes. This strategic approach not only ensures more targeted and efficient exploration but also enhances the overall effectiveness of the training. The decision-making network is thus trained more effectively on these classic behaviors, leading to more reliable and accurate training outcomes. This two-stage training process inherent in EADRL marks a prominent advancement in ensuring the effectiveness of training results while simultaneously reducing the complexity and uncertainty of environmental exploration.

In addition, EADRL has a certain promotion of autonomous navigation technologies. It brings intelligent surface vessels and self-driving cars the ability to autonomously navigate without human intervention. Especially for the intelligent surface vessel, EADRL allows intelligent surface vessels autonomously cruise, transport, and survey in scenarios where intelligent surface vessels and manned vessels coexist. We have proved that EADRL can be applied to shipping, and has broad prospects in maritime supervision, scientific research, aquaculture, marine resources exploitation, water environment detection and other fields. And potential advantages include cutting down the cost of cargo transportation, reducing the risk of the transnational transmission of infectious diseases, improving navigation efficiency, and saving manpower. Intelligent surface vessels abide by common rules with human-crewed vessels, which would liberate manpower and avoid losses resulting from personnel operation errors, which exceedingly promotes the process of autonomous navigation from theory to practice. Moreover, our proposed method can provide a reference for other unmanned system control. Furthermore, the proposed staged training mechanism is suitable for resolving DRL tasks with complex navigation state space and action space. The development of intelligent vehicles autonomous navigation technology will certainly promote the process of the industrial intelligent revolution.

Overall, EADRL has accomplished autonomous navigation of intelligent surface vessels and self-driving cars. It improves the training stability of the decision-making network and simplifies the dimension of input states. Experiment results verified its effectiveness on natural supply vessels and self-driving cars, providing a vital reference and evidence for applying our EADRL in practice. The proposed method has the adaptability to complex and unknown environments, has the ability of self-evolution, and can be updated in the application, which saves the cost of accumulating datasets. In the future, we will use more DRL verification tasks to demonstrate the efficiency improvement of EADRL on tasks with complex states and action space.

## Methods

In this work, we (1) propose an EADRL to achieve autonomous navigation of intelligent surface vessels and self-driving cars; (2) use the function learning stage and the optimization learning stage to explore state space and train the decision-making network in stages, where a task-adaptive observed behavior classification technique is presented in the function learning stage, and the function learning stage acts as a foundation of optimization learning stage; (3) develop a test environment of autonomous navigation for intelligent surface vessels, and the experimental results validate the effectiveness of our method for autonomous navigation of intelligent surface vessels and self-driving cars.

In the following paragraphs, we present EADRL architecture and functional modules, including the function learning stage, the optimization learning stage, and the observed behavior classification technique. (The implementation details of EADRL are shown in Supplementary Note 4.).

### EADRL architecture

We found that in the realm of reinforcement learning tasks, a notable overlap in features exists within the state space and action space, which can impede the environment-explored efficiency of the decision-making network. It is particularly noticeable that if an observed state yields a low reward, similar states are likely to produce similar outcomes. This insight has led us to the strategy of selecting ’classic behavior states’ to represent clusters of similar observed states, thereby streamlining the complexity of state space and action space (Details can be found in Supplementary Note 2 and Supplementary Fig. 1).

The proposed EADRL consists of the function learning stage and optimization learning stage. In the function learning stage, EADRL uses the observed behavior classification technique to carry out a rough exploration of observation states to find the classic observed behavior states and learns essential functions. The classic observed behavior states are extracted from observed states. In the optimization learning stage, the decision-making network delicately explores observed states based on the knowledge learned in the function learning stage. The function learning stage is conducive to the decision-making network to explore the correct policy gradient descent direction, and the optimization learning stage can optimize the decision-making network parameters through delicate exploration and learning. After training, the decision-making network can output reasonable action with inputting environment states. Compared to D2RL^1^ filtering critical states based on a comprehensive exploration of the state space, EADRL prunes the state space and action space during decision-making network training (Details can be found in Supplementary Fig. 11). Moreover, we employed the motion models^34^ of intelligent surface vessels and self-driving cars to perform the output action, and built some adverse scenarios to assist decision-making networks training for intelligent surface vessels and self-driving cars (Details are shown as Supplementary Figs. 9–10).

### Function learning stage

In the function learning stage, environmental states exploration of EADRL is called classic behaviors exploration. The classic behaviors experience pool saves only the states and actions that can represent a series of observed states and actions. For classic behaviors exploration, the decision-making network chooses an action according to a classic behavior state. Moreover, the chosen action would be replaced by the classic behavior action. Then, classic behavior states and classic behavior actions can be accumulated in the classic behavior experience pool. Notable, the observed behavior classifier only works in the function learning stage. Classic behavior experiences are used to train the essential functions of the decision-making network. Happening collision, departing from the preset path without encounter, arriving at the destination, or finishing exploration will lead to task termination.

### Optimization learning stage

In the optimization learning stage, environmental states exploration of EADRL is called observation states exploration. For observation states exploration, the decision-making network randomly samples action in normal distribution to explore and save observation states. It is worth noting that classic behavior states are selected from the continuous observation states and can represent certain states with the same evaluation trend. Meanwhile, the decision-making network weights trained in the function learning stage are further trained and optimized in the optimization learning stage. The method delicately explores the environment state space in the optimization learning stage to find better decision-making network weights.

### Observed behavior classification technique

EADRL addresses the complexity of state spaces by segmenting the large state space into several sub-state spaces. In each sub-state space, a representative state is chosen to symbolize all states within that subset. This approach effectively compresses the originally vast state space, reducing the number of states that need to be processed during training. Selecting representative states for each sub-state space simplifies the training process of the reinforcement learning model. These representative states capture the characteristics of their respective sub-state spaces, enabling more effective generalization of the knowledge learned during training.

The process of selecting representative states essentially concentrates attention on key areas within the sub-state spaces, thereby reducing the expanse of exploration. This focus on representative states targets states that have a more important impact on the task, diminishing the need for exhaustive exploration of the entire state space during training. Effective segmentation of the state space and the selection of representative states allow for more targeted exploration. This focused exploration improves efficiency, enabling the agent to discover and learn important knowledge in the environment more quickly.

By reducing the number of environmental states and focusing on the area of exploration, EADRL lowers the uncertainty in the exploration process. The agent explores the known representative states more intensively, thereby enhancing the predictability and stability of the exploration process.

### Supplementary information

Supplementary Information

## Data Availability

The scenario data used for training and testing the EADRL is available on Figshare^35^.

## Code availability

All the training and testing codes used in this study are available within the Article. Original codes for training and testing the decision-making network, and example codes for executing autonomous navigation of intelligent surface vessels and self-driving cars are available on Zenodo^36^.
