# Author Correction: Stable training via elastic adaptive deep reinforcement learning for autonomous navigation of intelligent vehicles

**DOI:** 10.1038/s44172-024-00195-3

**Published:** 2024-03-18

**Authors:** Yujiao Zhao, Yong Ma, Guibing Zhu, Songlin Hu, Xinping Yan

**Affiliations:** 1 State Key Laboratory of Maritime Technology and Safety, Wuhan University of Technology, Wuhan, China; 2 School of Navigation, Wuhan University of Technology, Wuhan, China; 3 National Engineering Research Center for Water Transport Safety, Wuhan University of Technology, Wuhan, China; 4 Chongqing Research Institute, Wuhan University of Technology, Chongqing, China; 5 Sanya Science and Education Innovation Park, Wuhan University of Technology, Sanya, China; 6 Marine College, Zhejiang Ocean University, Zhoushan, China; 7 Institute of Advanced Technology, Nanjing University of Posts and Telecommunications, Nanjing, China; 8 Intelligent Transportation Systems Research Center, Wuhan University of Technology, Wuhan, China

**Keywords:** Engineering, Mathematics and computing

Correction to: *Communications Engineering* 10.1038/s44172-024-00182-8, published online 26 February 2024

In this article the author name Xinping Yan was incorrectly written as Xinpin Yan. The original article has been corrected.

