# Supplementary material for: Stable training via elastic adaptive deep reinforcement learning for autonomous navigation of intelligent vehicles
Source: Commun Eng. 2024 Feb 26;3:37. doi: 10.1038/s44172-024-00182-8 (PMC10955858; doi:10.1038/s44172-024-00182-8)
Supplement: Supplementary file 1 — Supplementary Information [file 44172_2024_182_MOESM1_ESM.pdf]

Supplementary Information for

Stable training via elastic adaptive deep reinforcement learning for autonomous navigation of intelligent vehicles

Yujiao Zhao[1,2,3,4,5], Yong Ma[1,2,3,4,5 ✉], Guibing Zhu[6], Songlin Hu[7] & Xinping Yan[1,3,8 ✉]

March 7, 2024

[1] State Key Laboratory of Maritime Technology and Safety, Wuhan University of Technology, Wuhan, China. [2] School of Navigation, Wuhan University of Technology, Wuhan, China. [3] National Engineering Research Center for Water Transport Safety, Wuhan University of Technology, Wuhan, China. [4] Chongqing Research Institute, Wuhan University of Technology, Chongqing, China. [5] Sanya Science and Education Innovation Park, Wuhan University of Technology, Sanya, China. [6] Marine College, Zhejiang Ocean University, Zhoushan, China. [7] Institute of Advanced Technology, Nanjing University of Posts and Telecommunications, Nanjing, China. [8] Intelligent Transportation Systems Research Center, Wuhan University of Technology, Wuhan, China. ✉ email: myongdl@whut.edu.cn; xpyan@whut.edu.cn

Contents

1 Supplementary Note 1: Uncertain stability and complex tasks in navigation of autonomous vehicles 2

2 Supplementary Note 2: Elastic adaptive deep reinforcement learning in a more general sense 3

3 Supplementary Note 3: Detailed verification results 4

4 Supplementary Note 4: Implementation details 13

1 Supplementary Note 1: Uncertain stability and complex tasks in navigation of autonomous vehicles

Deep Reinforcement Learning (DRL) has made significant strides, showcasing its prowess in complex tasks ranging from mastering video games to steering intelligent surface vessels and autonomous vehicles. Despite these impressive feats, DRL grapples with two fundamental challenges that hinder its broader application: uncertain stability and the complexity of tasks [1]. These challenges are deeply rooted in the intricacies of exploring high-dimensional environments and the limitations of empirical data quality. This introduction aims to unravel these challenges, emphasizing their impact on the evolution of DRL, especially in the navigation of intelligent surface vessels and the operation of self-driving cars.

Uncertain stability. Uncertain stability remains a primary concern in DRL, with neural networks being the backbone for approximating complex functions. However, their sensitivity to training data quality often leads to unstable learning processes. Such instability can cause drastic variations in learning outcomes, making reliable convergence a daunting task. For instance, Ref. [2] introduces a curiosity-driven exploration strategy to bolster the stability of dynamics models, enhancing intrinsic rewards in DRL. Furthermore, Ref. [3] explores enhancing DRL agent robustness through a reward poisoning adversarial attack method.

The non-stationary nature of reinforcement learning environments exacerbates this challenge. As these environments evolve, previously effective policies may become obsolete, necessitating continual adaptation and relearning by DRL agents. This dynamism, coupled with the exploration-exploitation dilemma, complicates the stability of DRL, especially in applications like intelligent surface vessel navigation and autonomous driving, where environmental dynamics are constantly shifting. Therefore, there is an urgent need for an efficient and stable exploration strategy to solve this kind of problem.

Complex tasks. DRL has been instrumental in addressing complex tasks characterized by intricate decision-making and high-dimensional state spaces. Ref. [4] explores reducing search space in high-dimensional feature selection problems, a vital aspect of complex task management. However, the sheer complexity of these tasks presents significant hurdles. Agents must navigate vast action and state spaces to develop effective strategies, a process that can be prohibitively time-consuming and computationally intensive [5,6]. Ref. [7] explores the importance of curiosity-driven learning for human learning behavior, providing critical references for the development of curiosity-driven exploration methods in DRL.

Sparse and delayed rewards in complex tasks create the ”credit assignment problem,” where determining the impact of specific actions on outcomes becomes increasingly challenging. This problem is magnified in environments with unknown or stochastic dynamics, common in scenarios like autonomous surface vessel navigation. Ref. [8] presents a Go-Explore structure to enhance the learning ability of DRL for tasks with sparse and deceptive rewards, ensuring training efficiency and stability. Ref. [9] tries to solve the problem learning from delayed rewards using the specific memories to credit past actions.

Environmental uncertainties. In DRL, environmental uncertainties pose additional challenges, often manifesting as sensor noise, perceptual ambiguities, or incomplete data [10]. In the context of autonomous navigation, sensor inaccuracies due to adverse weather or hardware limitations can significantly impact data quality. Many researchers have made outstanding attempts to address environmental uncertainties. Ref. [11] addressed the potential perception uncertainty of autonomous cars from the sensor noises, fuzzy features, and unfamiliar inputs by combining the Monte Carlo dropout method and distributed value function. Ref. [12] addresses autonomous collision avoidance in surface vessels by integrating an efficient environmental state approximation method, utilizing prior knowledge in ship collision avoidance. Similarly, Ref. [13] offers an exhaustive review of collision avoidance navigation technologies for surface vessels, highlighting the integration of diverse approaches to enhance performance. Ref. [14] focuses on collision avoidance for unmanned surface vessels, specifically tackling environmental, observational, and decision-making uncertainties.

These uncertainties can severely affect the decision-making capabilities of DRL agents, as they struggle to discern between environmental noise and actual changes. Stable learning algorithms capable of adapting to such noisy data and making informed decisions amidst ambiguities are crucial in overcoming these challenges.

Uncertain stability and complex tasks stand as formidable obstacles in the advancement of DRL, particularly in high-dimensional environments like intelligent surface vessel navigation and autonomous vehicle control. Overcoming these challenges requires innovative algorithms, enhanced exploration strategies, and robust techniques to manage the uncertainties inherent in empirical data. Addressing these issues is pivotal for harnessing the full potential of DRL in practical, real-world applications.

2 Supplementary Note 2: Elastic adaptive deep reinforcement learning in a more general sense

Referring to the deep reinforcement learning methods, the state space and action space define the domains of input states and output actions of the decision-making network, respectively. During the training environment exploration, the state space and action space can be divided into several disjoint subsets to improve the exploration efficiency.

Referring to the deep reinforcement learning methods, the state space and action space define the domains of input states and output actions of the decision-making network, respectively. During the training environment exploration, the state space and action space can be divided into several disjoint subsets to improve the exploration efficiency.

$$\begin{aligned} \mathbb{S} &= \bigcup_{1\leq i\leq C_S} \mathbb{S}_i, \bigcap_{1\leq i\leq C_S} \mathbb{S}_i = \emptyset, \mathbb{S}_i \subseteq \mathbb{S} \\ \mathbb{A} &= \bigcup_{1\leq j\leq C_A} \mathbb{A}_j, \bigcap_{1\leq j\leq C_A} \mathbb{A}_j = \emptyset, \mathbb{A}_j \subseteq \mathbb{A} \end{aligned} \tag{1}$$

where $\mathbb{S}$ and $\mathbb{A}$ donate universal sets of the state space and action space, respectively. C_S and C_A represent the numbers of subsets.

Generally, the optimization of the weights of the decision-making network can be achieved through approximate gradient descent, the gradient can be approximated as

$$\nabla_\theta \mathbb{E}_\pi[f_\theta(x)] \approx \frac{1}{n}\sum_{k=1}^{n} \nabla_\theta f_\theta(x_k) \tag{2}$$

Therefore, the training process of deep learning problem can be formulated as a parameter correction process,

$$\theta_{\max \mathbb{E}_\pi[f_\theta(x)]} = \theta_{init} + \sum_{k=1}^{N_\mathbb{S}} \nabla_\theta \mathbb{E}[q_\theta(s_k, a_k)] \tag{3}$$

where $\theta_{[.]}$ is the parameters of the decision-making network, θ_{init} is the initial parameters. $N_\mathbb{S}$ denotes the sample number of the state space. Specifically, according to the policy gradient theorem, the policy gradient of DRL methods can be estimated as

$$\widehat{\nabla J(\theta)} = \hat{q}_\pi(s,a)\frac{\nabla\pi(a \mid s,\theta)}{\pi(a \mid s,\theta)} \tag{4}$$

where $q_\pi(s,a)$ denotes the state-action value, s and a are samples of the state and action under the policy, $\hat{q}_\pi(s,a)$ is an unbiased estimation of $q_\pi(s,a)$.

For the EADRL approach, we propose to simplify the environment exploration and training difficulty through the division of the state space and action space. In Equation 5, s_c^i and a_c^j represent the representative classic state of subspace $\mathbb{S}_i$ and the representative classic action of subspace $\mathbb{A}_j$, respectively.

$$\exists {s^i}_c \in \mathbb{S}_i, v_\pi({s^i}_c) = \frac{\sum\limits_{s^i \in \mathbb{S}_i} v_\pi(s^i)}{N_{\mathbb{S}_i}}, \exists {a^j}_c \in \mathbb{A}_j, q_\pi(s,{a^j}_c) = \frac{\sum\limits_{a^j \in \mathbb{A}_j} q_\pi(s,a^j)}{N_{\mathbb{A}_j}} \tag{5}$$

Therefore, the policy gradient of EADRL can be formulated as

$$\widehat{\nabla J(\theta)} = \sum_{i \le C_S} \sum_{j \le C_A} \hat{q}_\pi(s^i, a^j) \frac{\nabla \pi(a^j \mid s^i, \theta)}{\pi(a^j \mid s^i, \theta)}$$
$$= \sum_{i \le C_S} \sum_{j \le C_A} N_{\mathbb{S}_i} N_{\mathbb{A}_j} \hat{q}_\pi(s_c^i, a_c^j) \frac{\nabla \pi(a_c^j \mid s_c^i, \theta)}{\pi(a_c^j \mid s_c^i, \theta)} \quad (6)$$

Thus, the average policy gradient of EADRL can be

$$\widehat{\nabla J(\theta)} \approx q_\pi(s_c^i, a_c^j \mid s_c^i \in \mathbb{S}_i, a_c^j \in \mathbb{A}_j) \frac{\nabla \pi(a^j \mid s^i, \theta)}{\pi(a \mid s, \theta)} \quad (7)$$

where, s^i and a^j represent the state in subspace $\mathbb{S}_i$ and the action in subspace $\mathbb{A}_j$, respectively.

Finally, we define a state s_c^i and an action a_c^j as classic state and action if

$$\hat{q}_\pi(s_c^i) = \frac{\sum_{s^i} q(s^i)}{N_{\mathbb{S}_i}}, s_c^i, s^i \in \mathbb{S}_i,$$
$$\hat{q}_\pi(s, a_c^j) = \frac{\sum_{a^j} q(s, a^j)}{N_{\mathbb{A}_j}}, a_c^j, a^j \in \mathbb{A}_j \quad (8)$$

where $\hat{q}_\pi(s_c^i)$ denotes the state value estimation, $\hat{q}_\pi(s, a_c^j)$ denotes the state-action value estimation, s_c^i, a_c^j are sample of the state subset $\mathbb{S}_i$ and the action subset $\mathbb{A}_j$, respectively. $N_{\mathbb{S}_i}$ is the number of elements in $\mathbb{S}_i$, and $N_{\mathbb{A}_j}$ is the number of elements in $\mathbb{A}_j$.

As shown in Supplementary Figure 1, our EADRL selects a set of classic behaviour states, where each classic behaviour state individually represents all states of one subspace.

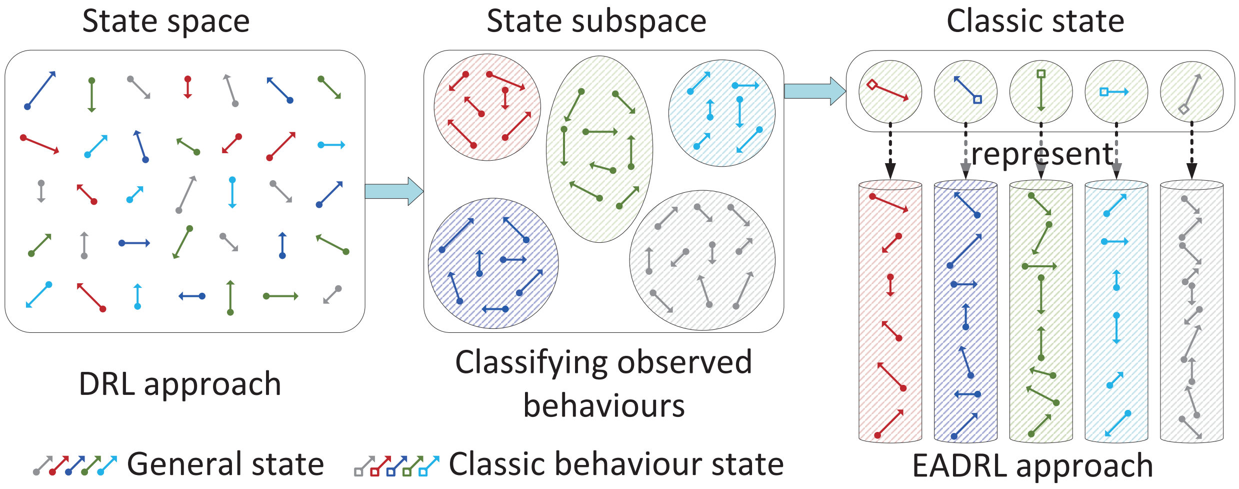

Supplementary Figure 1: **Illustration of the process of pruning state space through observed behaviour classifier.**

3 Supplementary Note 3: Detailed verification results

Intelligent surface vessel scenarios. We constructed four scenarios in the water near the Singapore strait to demonstrate that our EADRL allows intelligent surface vessels to navigate in multi-vessel encounter situations. A supply vessel with a displacement of 1,000 tons was selected as a prototype of the intelligent surface vessel in the following four scenarios.

- Scenario I: Two intelligent surface vessels navigate in the clear channels.

- Scenario II: An intelligent surface vessel navigates in the waters near the anchorage. Some passing surface vessels waiting to enter or depart the anchorage may appear in the channel where the intelligent surface vessel is, and the intelligent surface vessel needs to take reasonable actions to avoid passing surface vessels.
- Scenario III: Three intelligent surface vessels navigate in complex encounter situations. The collision avoidance situations including head-on situation, crossing situation, and over-taking situation occur continuously in the navigation process. The three intelligent surface vessels have to perform collision-avoidance behaviours to avoid each other.
- Scenario IV: Intelligent surface vessels autonomously navigate in a realistic multi-vessel encounter situation that happened in early March 2022 near the Eastern Boarding Ground of Singapore. The nearest encounter distance was only 1.3 times that of the length of the vessel, which had seriously violated the safe domain of other vessels and threatened navigation safety.

To facilitate performance evaluation of autonomous navigation for EADRL, we present detailed comparison results with line-of-sight (LOS) [15], pure pursuit (PP) [16], and artificial potential field (APF) [17] in Scenarios I, II, III and IV. Comparison trajectories of intelligent surface vessels in Scenarios I, II, III and IV are shown in Supplementary Figures 2, 3, 4, 5. Quantitative comparison results using different evaluation metrics, including velocities, headings, cross-track errors, along-track angle errors and encounter distances of intelligent surface vessels, are summarized in Supplementary Figure 6.

EADRL allows intelligent surface vessels to autonomously navigate in clear channels. Scenario I illustrates that two intelligent surface vessels autonomously navigate in their channels (Supplementary Figure 2). The preset paths were indicated by connecting path points, accorded with the navigation practice. The trajectories comparison of intelligent surface vessels using EADRL, LOS, PP and APF are shown in Supplementary Figure 2. Quantitative results using different evaluation metrics are summarized in Figure 6a, including velocity, heading, cross-track error, and along-track angle error. PP and APF yield similar performance for the various evaluation metrics in autonomous navigation of intelligent surface vessels, and LOS performs better than PP and APF. By comparison, it can be found that EADRL yields better performance than other methods in terms of velocity, heading maintenance, cross-track error, and along-track angle error. Even when intelligent surface vessels navigate near path points, the decision-making network has learned to eliminate cross-track errors by deceleration and turning.

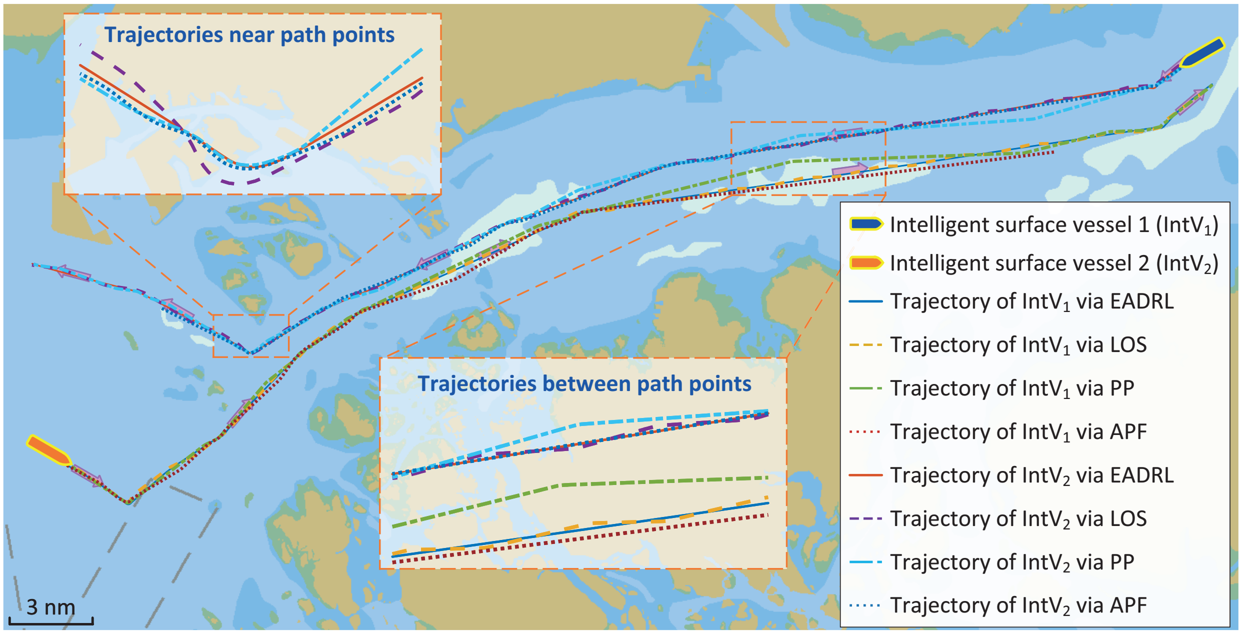

Supplementary Figure 2: **Trajectories of intelligent surface vessels using elastic adaptive deep reinforcement learning (EADRL), line-of-sight (LOS), pure pursuit (PP) and artificial potential field (APF) in Scenario I.** The blue vessel and orange vessel are intelligent surface vessels. The pink dash lines are the channel boundary, and the pink arrows indicate the channel direction. "nm" means nautical mile. Two intelligent surface vessels autonomously navigate in the channels, and EADRL, LOS, PP and APF algorithms are used to navigate the vessels. Trajectories of LOS and PP are pretty different from the preset path. The trajectories of intelligent surface vessels via EADRL and APF are better than LOS and PP. Especially, the trajectories of intellignet vessels via EADRL perform minor cross-track errors than those three methods.

EADRL navigates the intelligent surface vessel in the water near the anchorage. When the intelligent surface vessel autonomously navigates near the anchorage area, it avoids collisions with vessels waiting to enter or depart the anchorage. Those vessels maintain low speeds and cannot actively avoid collisions. In addition, those vessels may occupy the channel for a long while waiting, and it will waste a lot of time waiting for the channel to be clear. Our EADRL successfully navigates intelligent surface vessels to bypass other vessels in various situations.

In Scenario II, an intelligent surface vessel followed a preset path in the channel near the East anchorage of Singapore. The trajectories comparison of intelligent surface vessels using EADRL and APF are shown in Supplementary Figure 3. The intelligent surface vessel performed reasonable and safe collision avoidance behaviours using our EADRL. When the intelligent surface vessel detected that several vessels occupied its channel, it decelerated and turned to starboard to avoid those vessels that occupied lanes. After bypassing the first vessel, the intelligent surface vessel detected the second and performed collision avoidance behaviours again.

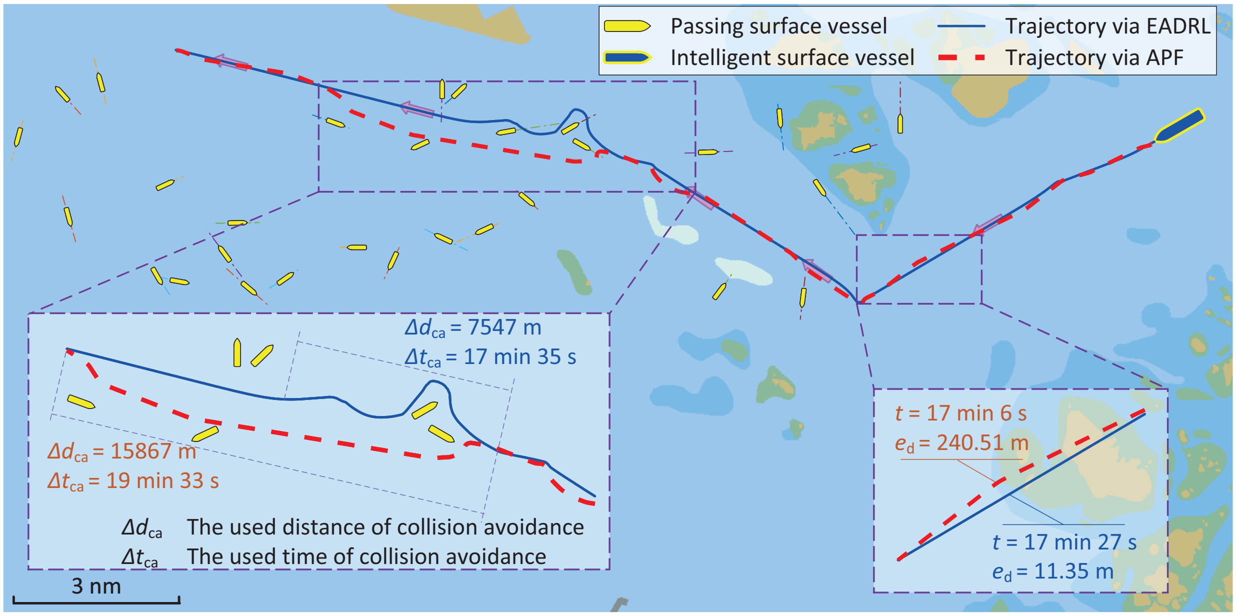

Supplementary Figure 3: **Trajectories of the intelligent surface vessel using elastic adaptive deep reinforcement learning (EADRL) and artificial potential field (APF) in Scenario II.** "nm" means nautical mile. An intelligent surface vessel autonomously navigates via EADRL and APF, and the intelligent surface vessel can bypass the passing surface vessels to avoid collision when the passing surface vessels appear in the channel. The trajectory of the intelligent surface vessel via EADRL is closer to the preset path. Meanwhile, EADRL controls intelligent surface vessel turning to starboard to pass under COLREGs, while APF controls intelligent surface vessel turning to port without COLREGs. The intelligent surface vessel uses less distance and time to achieve collision avoidance via EADRL than APF.

EADRL achieves intelligent surface vessels to autonomously navigate under the interference of other vessels. Intelligent surface vessels need to avoid collisions with passing surface vessels frequently in multi-vessel encounter scenarios. The rationality of collision avoidance behaviours taken by intelligent surface vessels has significant influence in complex collision avoidance situations. A complicated encounter situation between three intelligent surface vessels is shown in Supplementary Figure 4. As shown in Supplementary Figure 4a, by EADRL, $IntV_1$ formed a head-on encounter situation with $IntV_2$, and $IntV_3$ created crossing encounter situations with $IntV_1$ and $IntV_2$, respectively. Then, $IntV_3$ performed deceleration behaviour first in the encounter situation, and then $IntV_2$ and $IntV_1$ decelerated successively. Later, $IntV_1$ turned to the starboard to avoid $IntV_2$. After that, $IntV_3$ turned to starboard to avoid $IntV_1$ and $IntV_2$. And then, all three intelligent surface vessels finished collision avoidance and continued to follow their preset paths. In contrast, intelligent surface vessels using APF have generated much unnecessary trajectory, resulting in poor collision avoidance effects.

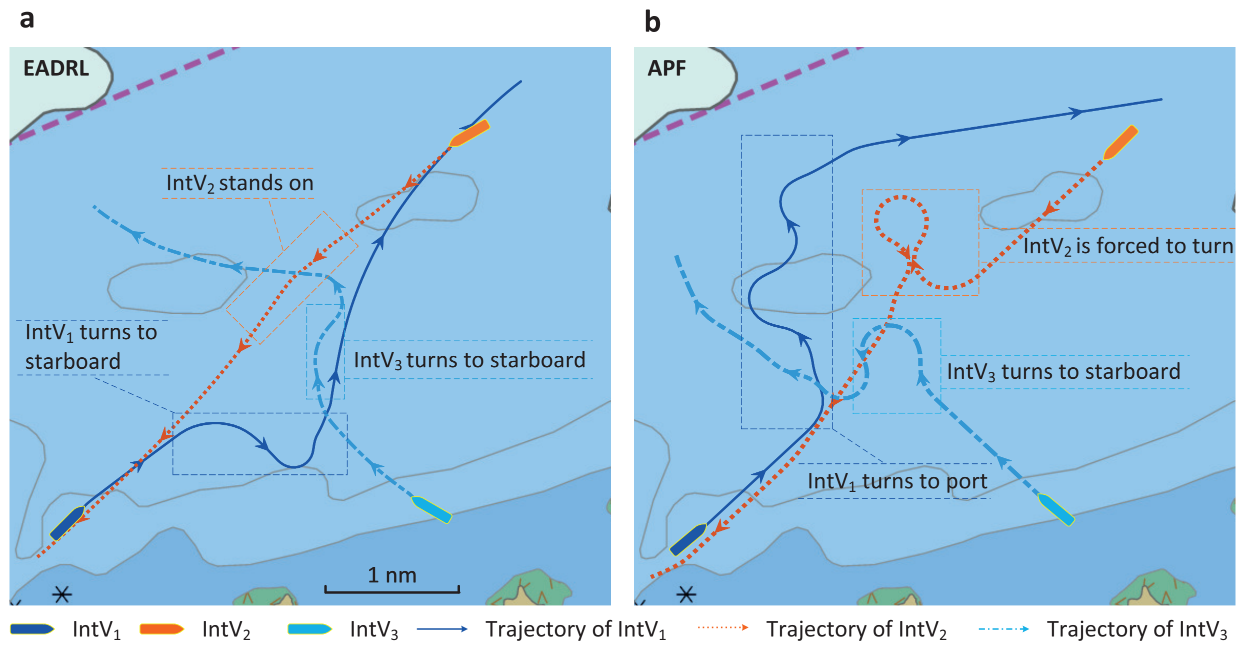

Supplementary Figure 4: **Trajectories comparison of three intelligent surface vessels using elastic adaptive deep reinforcement learning (EADRL) and artificial potential field (APF) in Scenario III. a, Trajectories of intelligent surface vessels using EADRL.** Three intelligent surface vessels form an urgent encounter situation. $IntV_1$ turned to starboard to give way to $IntV_3$ while $IntV_3$ turned to starboard to give way to $IntV_2$, following the COLREGs. $IntV_2$ sailed along the preset path as a stand-on vessel. "nm" means nautical mile. **b, Trajectories of intelligent surface vessels using APF.** The trajectories shows that three intelligent surface vessels avoid each other without rules. Trajectories of intelligent surface vessels are with long lengths, indicating that EADRL is more efficient than APF.

EADRL guarantees intelligent surface vessels to navigate in multi-vessel encounter situation. To further demonstrate the superiority of the EADRL, a comparative simulation was conducted with the APF algorithm in the scenario of multi-vessel encounters. Supplementary Figure 5 shows the compared trajectories of five intelligent surface vessels using EADRL and APF. In which, EADRL has advantages than APF in trajectory smoothness and steering timing selection.

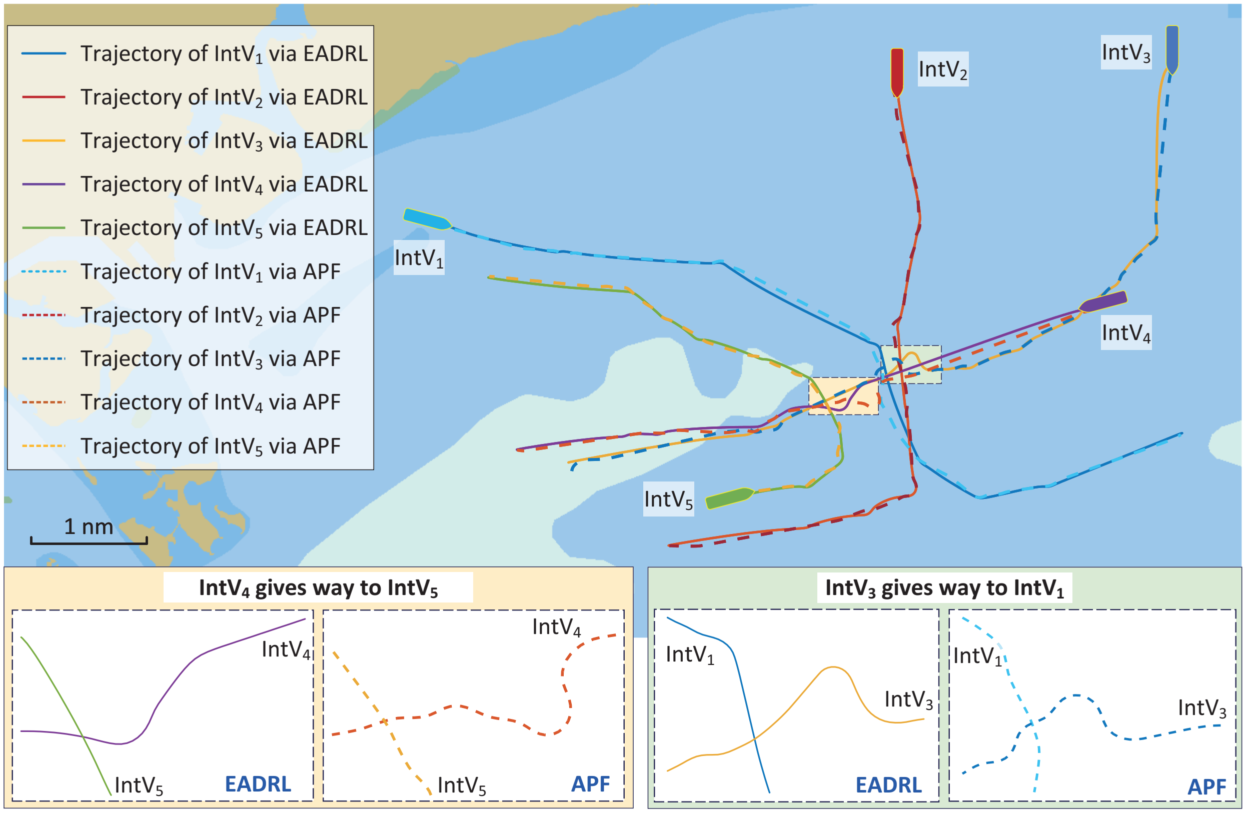

Supplementary Figure 5: **Trajectories comparison of five intelligent surface vessels using elastic adaptive deep reinforcement learning (EADRL) and artificial potential field (APF) in Scenario IV.** "nm" means nautical mile. Five intelligent surface vessels formed an urgent encounter situation. IntV_1 crossed the down-channel and entered the up-channel, IntV_2 crossed the down-channel and up-channel, IntV_3 and IntV_4 entered the down-channel and navigated in the down-channel, IntV_5 crossed the down-channel from the up-channel and departed from the channel. By means of APF, intelligent surface vessels followed preset paths and avoided collisions. However, in the process of collision avoidance, IntV_1 and IntV_3 got entangled. Under the action of EADRL, IntV_1 and IntV_3 avoided collision by turning to starboard and deceleration under COLREGs.

Supplementary Figure 6 shows quantitative comparison of autonomous navigation performance in intelligent surface vessel scenarios. As shown in Supplementary Figure 6a, all four method can maintain the velocities of intelligent surface vessels in [8, 9] $\text{m}\cdot\text{s}^{-1}$, [15.6, 17.5] knots. However, EADRL performed better than the others in the cross-track error, where IntV_1 followed the preset path with a cross-track error of average 35 m and maximum 368 m, and values of IntV_2 were 31 m and 312 m. The along-track angle error changes synchronously with the cross-track error, and it remains stable at around 0. Preset paths turn at the path points, resulting in velocities, cross-track errors and along-track angle errors fluctuating when intelligent surface vessels navigated near path points. Several metrics were used to quantitatively evaluate the performance of intelligent surface vessel autonomous navigation, containing velocity, heading, cross-track error, and along-track angle error (Supplementary Figure 6b). EADRL performs similarly to APF in velocity, heading, and along-track angle error but is significantly better than APF in terms of cross-track error. The velocity, heading, cross-track error and along-track angle error remained stable before encountering obstacles and fluctuated during collision avoidance. And, the encounter distances between intelligent surface vessels, headings, velocities, and along-track angle errors of intelligent surface vessels in Scenario III and Scenario IV are presented in Supplementary Figure 6c and d, respectively. Compared with APF, EADRL is generally larger in terms of the closet

envounter distance between intelligent surface vessels, and smaller in terms of trajectory length. The nearest encounter distance was five times the length of the intelligent surface vessel, and it was safe enough for the intelligent surface vessel.

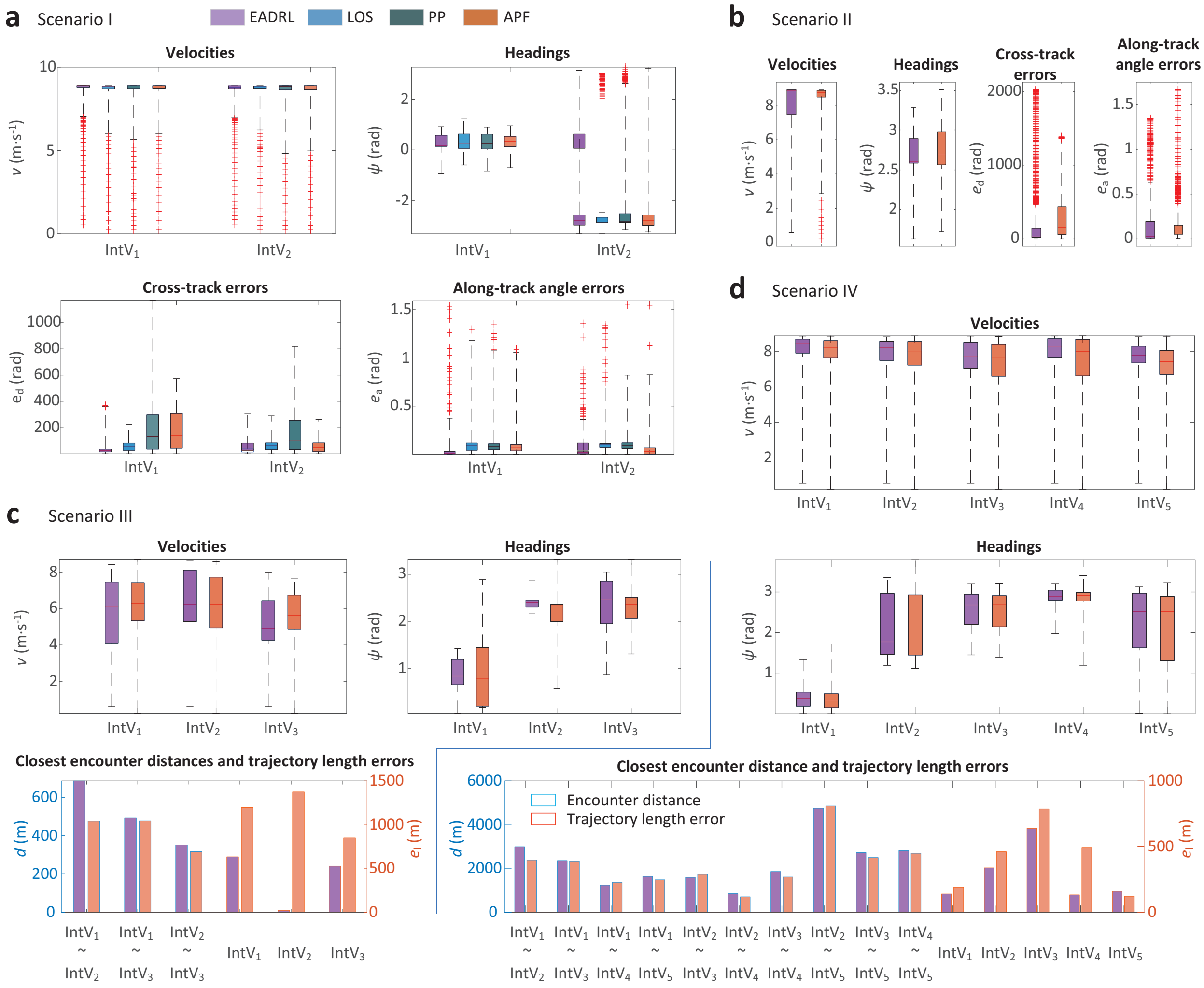

Supplementary Figure 6: **Quantitative comparison of autonomous navigation performance in intelligent surface vessel scenarios.** Numerical evaluation and various metrics (velocity, heading angle, cross-track error, along-track angle error, encounter distance, and trajectory length error) of autonomous navigation of intelligent surface vessels. The red central mark indicates the median, and the bottom and top edges of the box indicate the 25th and 75th percentiles, respectively. The whiskers extend to the most extreme data points not considered outliers, and the outliers are plotted individually using the '+' symbol. Compared with the other methods, elastic adaptive deep reinforcement learning achieves higher encounter distance and velocity and maintains lower cross-track error, along-track angle error, and trajectory length errors. Regarding autonomous navigation, minor cross-track and along-track angle errors, fast velocity, smooth heading angle, the more considerable encounter distance and shorter trajectory length correspond to the better performance.

EADRL drives self-driving car through crowded road. Supplementary Figure 7 illustrates image frames captured at various moments in the self-driving car scenario. In Supplementary Figure 7a, the self-driving car is shown navigat-

ing through an intersection by decelerating to yield to oncoming cars, successfully passing through the intersection. Supplementary Figure 7b demonstrates the self-driving car negotiating a right turn at an intersection while maintaining its lane. In Supplementary Figure 7c, the self-driving car autonomously applies brakes to yield to another car while being overtaken. Supplementary Figure 7d depicts the self-driving car overtaking another car during its travel.

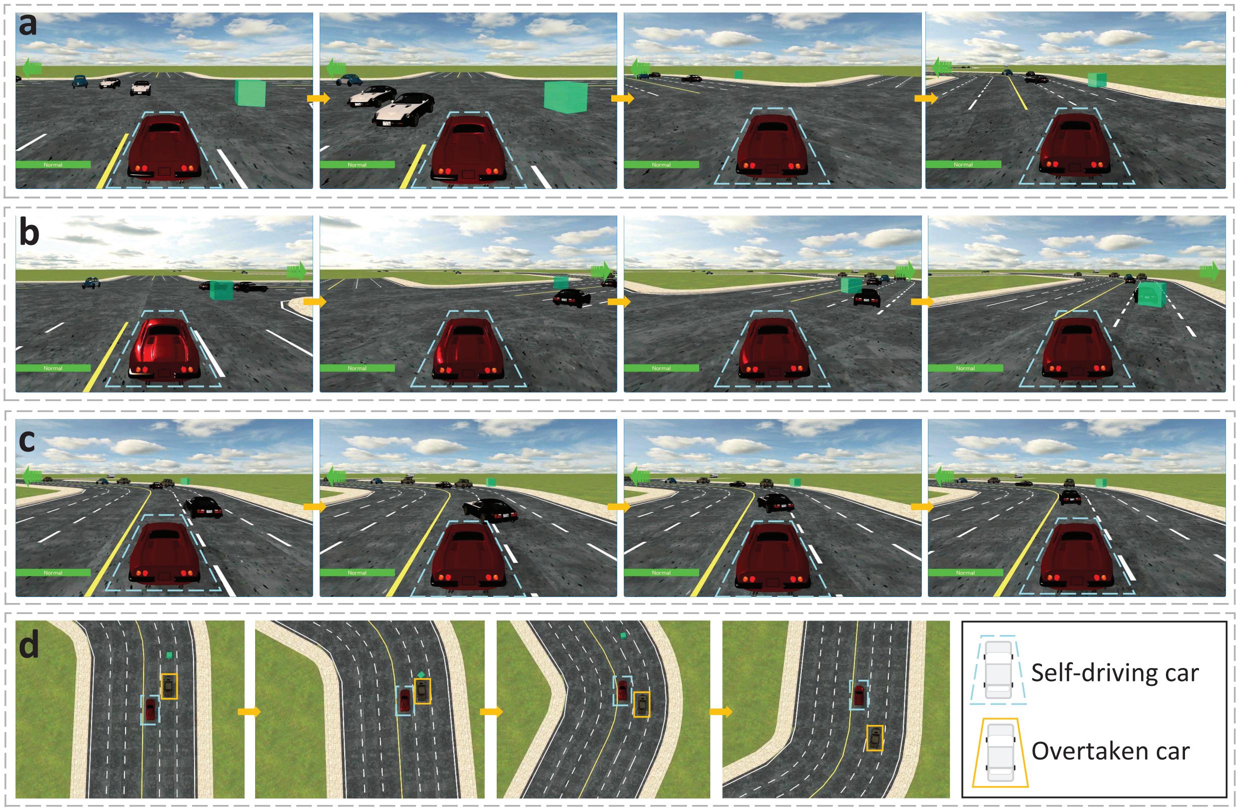

Supplementary Figure 7: **Frames in the self-driving car scenario. a, The self-driving car passes an intersection to the left with interference from other cars. b, The self-driving car passes an intersection to the right. c, The self-driving car is overtaken by other car. d, The self-driving car overtakes other car.**

Supplementary Figure 8 shows variation curves of the acceleration of self-driving car, angle-error between self-driving car and test lane, step reward, and distance between self-driving car and the left yellow continuous line in the self-driving car scenario. As shown in Supplementary Figure 8a, the acceleration of self-driving car has excellent responsiveness and smoothness. EADRL enables rapid and smooth acceleration and deceleration across different speed ranges. Simultaneously, Supplementary Figure 8b, c, d indicate that EADRL effectively achieves precise direction control, ensuring stable car travel on complex roads. This reduces unnecessary directional adjustments, enhancing driving smoothness and safety. Through continuous adjustment of the car position, EADRL can maintain an appropriate distance from the middle yellow line, avoiding collisions with other cars and contact with obstacles at the road edge. As a result, EADRL receives consecutive positive rewards.

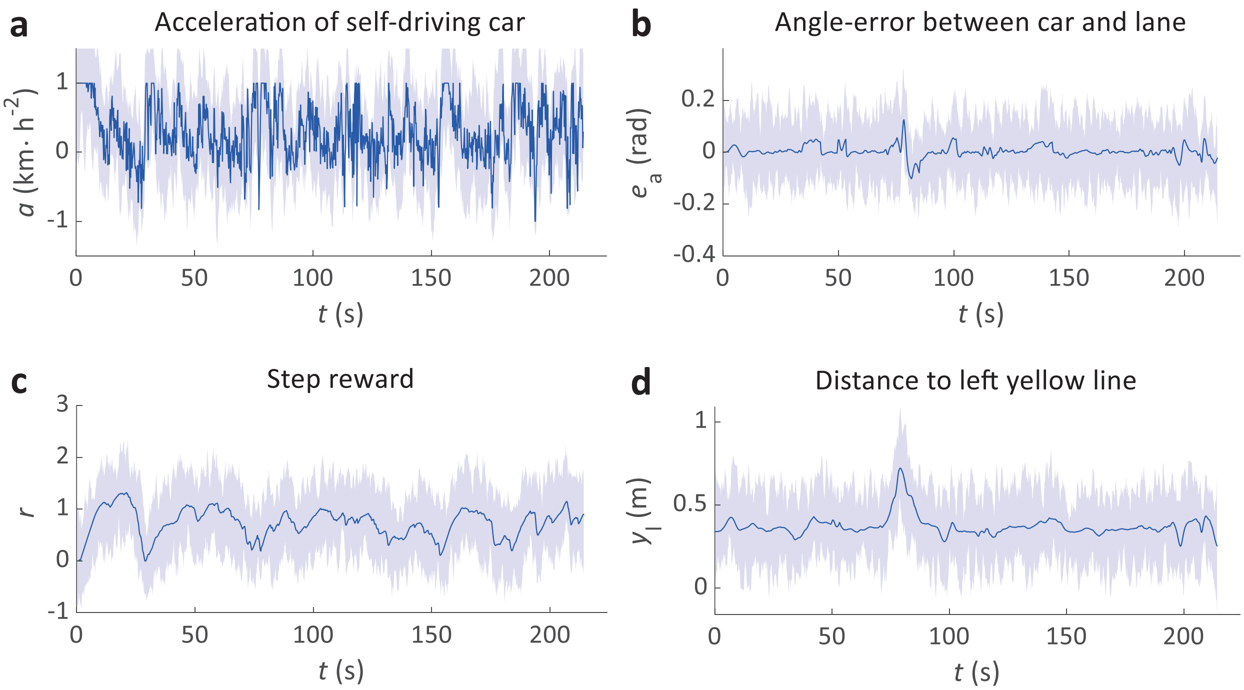

Supplementary Figure 8: **Qualitative results of elastic adaptive deep reinforcement learning in self-driving car scenario. a, Acceleration of self-driving car.** The light grey shaded region represents the 85% confidence level of acceleration of self-driving car. **b, Angle-error between self-driving car and test lane.** The light grey shaded region represents the 85% confidence level of angle-error between self-driving car and test lane. **c, Step reward.** The shaded areas in **c** represent the standard deviation. **d, Distance between self-driving car and the left yellow continuous line.** The light grey shaded region represents the 85% confidence level of distance between self-driving car and the left yellow continuous line.

4 Supplementary Note 4: Implementation details

Training environment of EADRL. EADRL was implemented using Tensorflow. The evaluation was based on a machine with a CPU (Intel i9-13900K) and a GPU (NVIDIA GeForce RTX 4090 24-GB RAM). The RMSprop optimizer with 1×10^{-4} and 1×10^{-3} learning rate was utilized for minimizing the loss of the actor network and critic network, respectively.

Reward functions of intelligent surface vessls. Considering complex situations in autonomous navigation, we designed a series of reward functions in the form of exponent function and first-order polynomial. In which the dependent variable of the first-order polynomial changes smoothly, the dependent variable of the exponent function changes sharply. The cross-track error is usually large in collision avoidance, a form of the first-order polynomial is adopted to the path following reward function to reduce the impact of path following reward on collision avoidance performance. In collision avoidance, the collision risk is higher as the distance between the intelligent surface vessel and the passing surface vessels smaller. To attract enough attention from the decision-making network to collision risk, an exponential form is adopted for the collision avoidance reward function. Eventually, a composite polynomial reward function integrating intelligent surface vessel motion factors, path following factors and collision avoidance factors is designed.

For the autonomous navigation of intelligent surface vessels, it is necessary to eliminate cross-track error and angle error between the heading of the intelligent surface vessel and the preset path. Thus, we designed a path following reward to inspire decision-making agents to learn to control intelligent surface vessels sailing on preset paths. We designed path following reward as Equations (9, 10).

$$r_{\text{ed}} = -\frac{e_{\text{d}}}{e_{\text{d}}^{\max}}, r_{\text{ea}} = -\exp^{\frac{-1}{2*e_{\text{a}}+b_{\text{ea}}}}, r_{\text{v}} = \frac{|\nu|}{|\nu|^{\max}} - 1 \tag{9}$$

$$r_{\text{pf}} = m_{\text{ed}} * r_{\text{ed}} + m_{\text{ea}} * r_{\text{ea}} + m_{\text{v}} * r_{\text{v}} \tag{10}$$

where r_{pf} is the path following reward. r_{ed} is the path-following cross-track error reward. r_{ea} is the path-following angle error reward. e_{d} is the cross-tracking error. $e_{\text{a}} \in [-\pi, \pi]$ is the angle error between intelligent surface vessel heading and preset path. $e_{\text{d}}^{\max}$ is the maximum cross-track error allowed in the path following. m_{ed}, m_{ea}, and m_{v} are weight of r_{ed}, r_{ea}, and r_{v}, respectively. b_{ea} is a bias to limit the codomain of r_{ea} in $[-1, 0]$. r_{ed} adopts a linear variation form rather than an exponential form. r_{ea} adopts the exponential variation form. The reward is normalized to improve the training efficiency of the decision-making network and reduce calculation.

For collision avoidance of intelligent surface vessels, the most important objective is to keep safe distances from passing surface vessels in different situations. We designed collision avoidance reward as Equations (11)-(13).

$$d_{\text{ca}}^{\min} = D_i, \; i = \arg\min\left(\frac{dis_{\text{IntV\_PasV}}}{\nu_{\text{REL}}^{\text{a}}}\right) \tag{11}$$

where $d_{\text{ca}}^{\min}$ is the distance between the closest vessel and the intelligent surface vessel. D is the set of distances between the intelligent surface vessel and all passing surface vessels. Set $dis_{\text{IntV\_PasV}}$ are distances between the intelligent surface vessel and passing surface vessels. $\nu_{\text{REL}}^{\text{a}}$ is the component of velocity ν_{REL} that along the line between the intelligent surface vessel and the passing vessel, and $\nu_{\text{REL}}^{\text{c}}$ is the component of velocity ν_{REL} that is perpendicular to the line between the intelligent surface vessel and the passing vessel. $\nu_{\text{REL}} = [\nu_{\text{REL}}^{\text{a}}, \nu_{\text{REL}}^{\text{c}}]$.

$$risk_{\text{ca}} = \begin{cases} kr_{\text{h}} & \text{if head-on} \\ kr_{\text{cg}} & \text{if crossing and give-way} \\ kr_{\text{cs}} & \text{if crossing and stand-on} \\ kr_{\text{o}} & \text{if overtaking} \end{cases}, 0 < kr_{\text{o}} < kr_{\text{cs}} < kr_{\text{cg}} < kr_{\text{h}} \leqslant 1 \tag{12}$$

$$r_{\text{ca}} = -risk_{\text{ca}} * \exp^{-m_{\text{ca}} * \frac{d_{\text{ca}}^{\min}}{d_{\text{s}}} + b_{\text{ca}}} \tag{13}$$

where, r_{ca} is collision avoidance reward, $risk_{\text{ca}}$ is coefficient indicating the risk of collision (Equation (6)). The exponential form is used to weaken the influence of other passing surface vessels on the intelligent surface vessel when it is far and strengthen that when it is close. Thus, it can keep distances between the intelligent surface vessel and other passing surface vessels outside the dangerous range.

The multi-task reward function is presented in Equation (14).

$$\begin{aligned} r_{\text{a}} =& k_{\text{pf}} * (m_{\text{ed}} * \frac{e_{\text{d}}}{e_{\text{d}}^{\max}} + m_{\text{ea}} * \exp^{\frac{-1}{e_{\text{a}}*2+b_{\text{ea}}}} \\ &+ m_{\text{v}} * (\frac{|\nu|}{|\nu|^{\max}} - 1)) \\ &- k_{\text{ca}} * risk_{\text{ca}} * \exp^{-m_{\text{ca}}*\frac{d_{\text{ca}}^{\min}}{d_{\text{s}}}+b_{\text{ca}}} \end{aligned} \tag{14}$$

where $k_{\text{pf}}, k_{\text{ca}} \in [0, 1]$ are training weight coefficients of path following and collision avoidance, respectively. r_{a} represents the reward.

Intelligent surface vessel scenarios. For the reward function, we used the following parameters $e_{\text{d}}^{\max} = 300$, $b_{\text{ea}} = \frac{1}{4}$, $|\nu|^{\max} = 12$, $m_{\text{ed}} = 1$, $m_{\text{ea}} = \frac{3}{2}$, $m_{\text{v}} = \frac{1}{2}$, $kr_{\text{h}} = -1$, $kr_{\text{cg}} = -1$, $kr_{\text{cs}} = -\frac{3}{4}$, $kr_{\text{o}} = -0.3$, $m_{\text{ca}} = \frac{1}{3}$, $b_{\text{ca}} = \frac{1}{3}$, $d_{\text{s}} = 228.6$, $k_{\text{pf}} = \frac{1}{3}$, and $k_{\text{ca}} = 1$. For the reward discount, we used $\gamma = 0.7$. For the training, the experience pool size we used $R = 10^5$, the batch size $B = 64$. We used the elliptical domain with a long axis of seven times the length of the vessel and a short axis of three times the length of the vessel as the safe domain of the vessel.

In this work, we take a supply vessel as the prototype of the intelligent surface vessel. The scale of the intelligent surface vessel is $(76.2, 15)$ m, the displacement is 1,000 tons. The acceleration of gravity $g = 9.81$ m·s^{-2}. The detailed parameters of the intelligent surface vessel are as below.

$$M = \begin{bmatrix} 6.7644 \times 10^6 & 0 & 0 \\ 0 & 1.1341 \times 10^7 & -3.4016 \times 10^6 \\ 0 & -3.4016 \times 10^6 & 4.4524 \times 10^7 \end{bmatrix}$$

$$D = \begin{bmatrix} 7.7071 \times 10^4 & 0 & 0 \\ 0 & 2.5468 \times 10^5 & -2.0342 \times 10^6 \\ 0 & -6.7258 \times 10^5 & 3.8501 \times 10^8 \end{bmatrix}$$

$$C = \begin{bmatrix} 0 & 0 & -11.3410v + 3.4016r \\ 0 & 0 & 6.7644u \\ 11.3410v - 3.4016r & -6.7644u & 0 \end{bmatrix} \times 10^6$$

Collision accidents of intelligent surface vessels are rare in the objective world, it is difficult to collect enough real data to train the decision-making network. Therefore, a repeatable collision scenario was constructed based on real accident data to collect training data. In the training process of intelligent surface vessels, a massive amount of adverse situation data (Supplementary Figure 9) is produced through human guidance to assist decision-making networks in learning autonomous collision avoidance function.

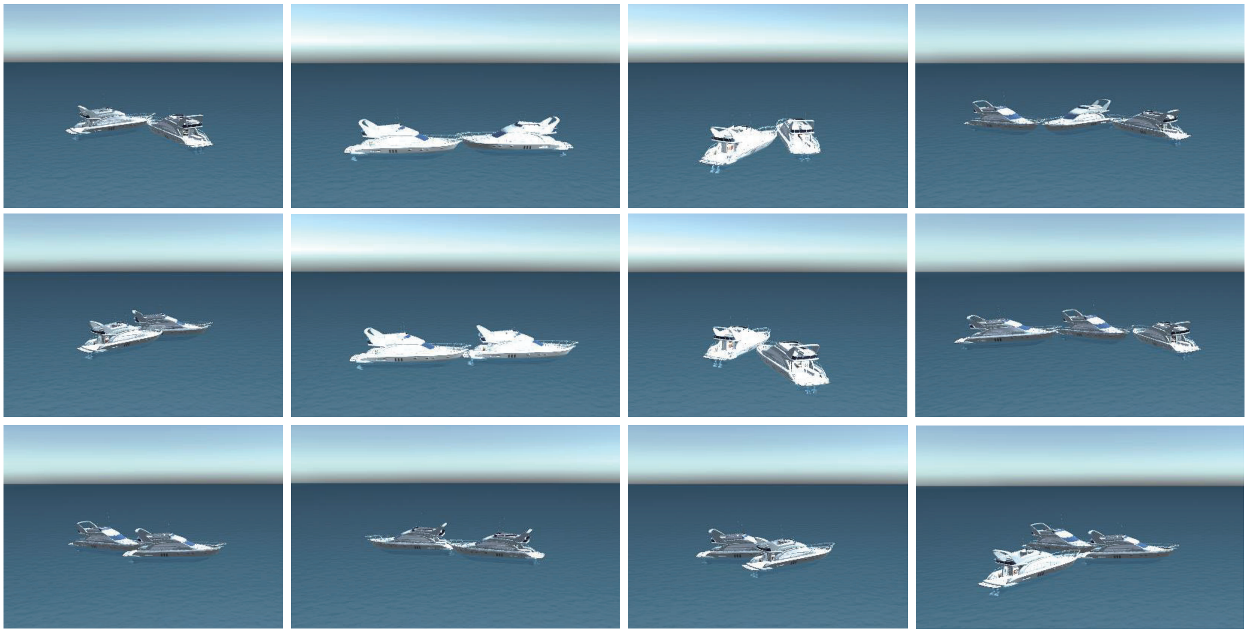

Supplementary Figure 9: **Adverse situations of intelligent surface vessel autonomous navigation scenarios.**

Self-driving car scenarios. The simulations of self-driving car scenarios are conducted through the MetaDrive environment [18]. The simulation benchmark is to navigate the self-driving car passing through straight lanes, curve lanes, intersections, and safely reach the destination through avoidance, overtaking, and other behaviours.

The training data of self-driving cars for evaluation are collected by randomly sampling the state s, action a and rewards r in a self-driving test environment to simulate the data in real-world driving. Among the training data, many critical data are specially treated, especially adverse situations of car driving and complex roads Supplementary Figure 10.

At the function learning stage, the decision-making network learns to output actions to play basic navigation behaviours to fulfil task objectives. Classic behaviours include going ahead, steering left, steering right, accelerating, decelerating, and braking.

Related outstanding research. Deep reinforcement learning holds immense potential in addressing autonomous vehicle navigation challenges, and research focused on enhancing the training efficiency of deep reinforcement learning is worthy of attention. Dense deep reinforcement learning (D2RL) [19] is an outstanding work in utilizing deep reinforcement learning methods to achieve safety validation of autonomous vehicles. D2RL filters critical states based on comprehensive exploration of the state space, reducing the proportion of uncritical state data and building a high-quality experience dataset. It addresses the issue of inefficient algorithm training due to the scarcity of high-value data in reinforcement learning tasks by increasing the density of rare data. D2RL contributed to construct a validation environment for autonomous driving algorithms.

In contrast, this work aims to achieve autonomous navigation for both self-driving cars and intelligent surface vessels, and our focus is more on the stability of the reinforcement learning training process. As shown in Supplementary Figure 11, EADRL prunes the state space and action space rather than experience dataset to optimize the exploration efficiency of state space and action space, during decision-making network training.

Worthy, the advantage of EADRL in training stability becomes even more apparent when compared with D2RL. This coincidence highlights the superior performance of EADRL in maintaining stable training outcomes. As shown in Supplementary Figure 12, the reward values of EADRL exhibit a stepped change, attributed to EADRL dividing

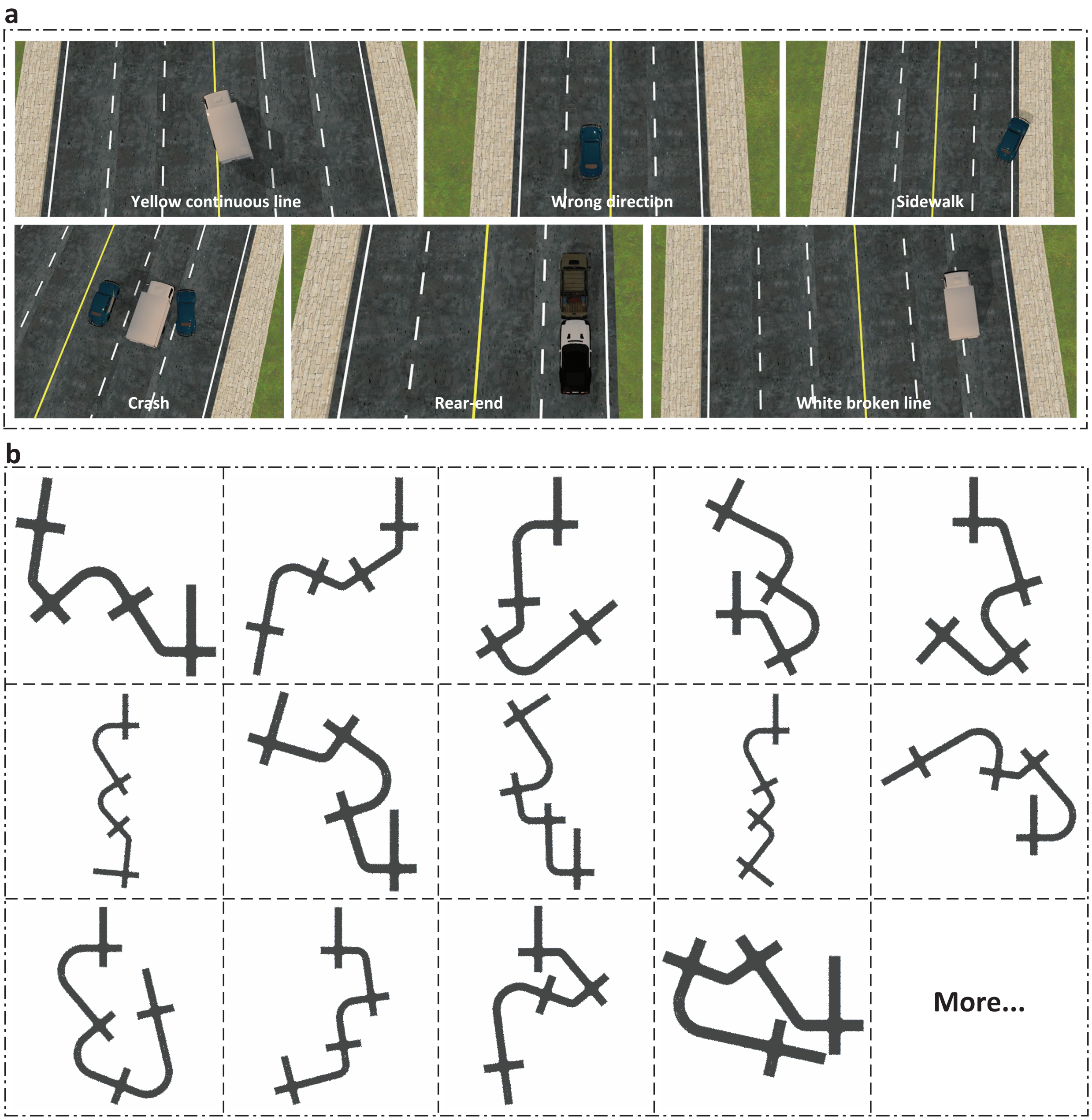

Supplementary Figure 10: **Training data used in self-driving car scenarios. a, Adverse situations. b, Training and test maps.**

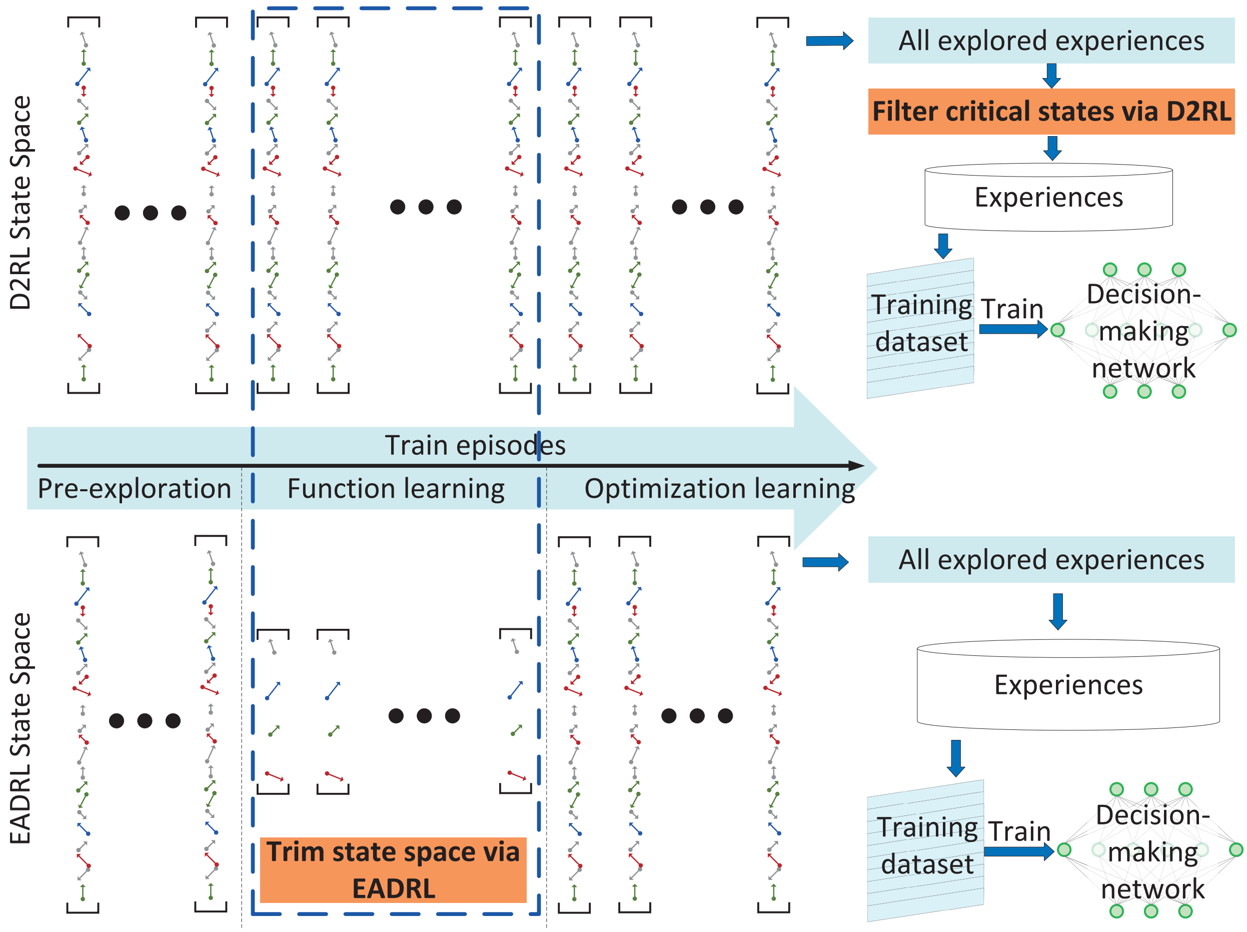

Supplementary Figure 11: **Comparation of training processes between dense deep reinforcement learning and elastic adaptive deep reinforcement learning.**

the training process into functional learning and optimization learning stages. EADRL employs an adaptive observed behaviour classification technique in the functional learning stage, while D2RL collects effective training experiences slower in the early stages, resulting in a slower initial learning pace. Consequently, EADRL learns higher reward values faster than D2RL in the function learning stage of training, and it shows more stable reward variations in its optimization learning stage.

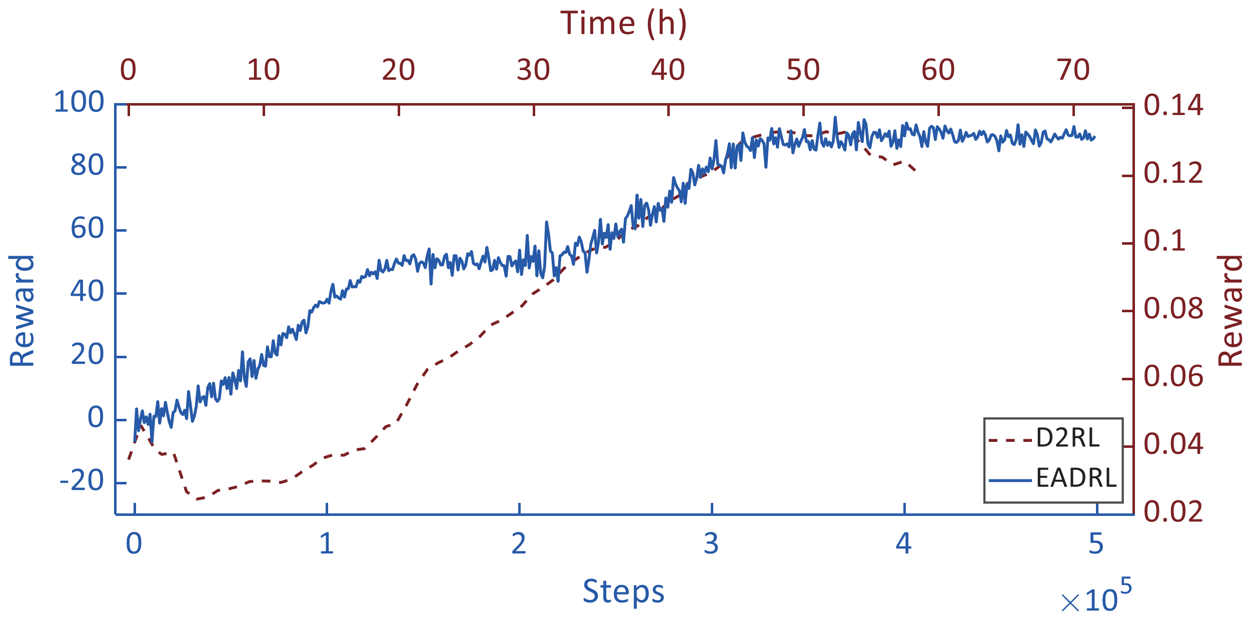

Supplementary Figure 12: **The comparison of rewards during the training process of dense deep reinforcement learning and elastic adaptive deep reinforcement learning.**

In conclusion, the D2RL addresses the challenge of having too few critical states for successful learning, making it significantly different from traditional DRL approaches. EADRL tackles the issues of training efficiency and stability, showcasing its performance in training stability compared to traditional DRL approaches.

Supplementary References

[1] Kim, D., Park, G. Y., O'Doherty, J. P. & Lee, S. W. Task complexity interacts with state-space uncertainty in the arbitration between model-based and model-free learning. *Nature Communications* **10**, 5738 (2019).

[2] Li, J., Shi, X., Li, J., Zhang, X. & Wang, J. Random curiosity-driven exploration in deep reinforcement learning. *Neurocomputing* **418**, 139–147 (2020).

[3] Cai, K., Zhu, X. & Hu, Z. Reward poisoning attacks in deep reinforcement learning based on exploration strategies. *Neurocomputing* **553**, 126578 (2023).

[4] Song, X.-F., Zhang, Y., Gong, D.-W. & Gao, X.-Z. A fast hybrid feature selection based on correlation-guided clustering and particle swarm optimization for high-dimensional data. *IEEE Transactions on Cybernetics* **52**, 9573–9586 (2021).

[5] Yuan, K., Sajid, N., Friston, K. & Li, Z. Hierarchical generative modelling for autonomous robots. *Nature Machine Intelligence* **5**, 1402–1414 (2023).

[6] Eppe, M. *et al.* Intelligent problem-solving as integrated hierarchical reinforcement learning. *Nature Machine Intelligence* **4**, 11–20 (2022).

[7] Ten, A., Kaushik, P., Oudeyer, P.-Y. & Gottlieb, J. Humans monitor learning progress in curiosity-driven exploration. *Nature Communications* **12**, 5972 (2021).

[8] Ecoffet, A., Huizinga, J., Lehman, J., Stanley, K. O. & Clune, J. First return, then explore. *Nature* **590**, 580–586 (2021).

[9] Hung, C.-C. *et al.* Optimizing agent behavior over long time scales by transporting value. *Nature Communications* **10**, 5223 (2019).

[10] Negenborn, R. R. *et al.* Autonomous ships are on the horizon: here's what we need to know. *Nature* **615**, 30–33 (2023).

[11] Liu, J. *et al.* Pnnuad: Perception neural networks uncertainty aware decision-making for autonomous vehicle. *IEEE Transactions on Intelligent Transportation Systems* **23**, 24355–24368 (2022).

[12] Wang, C., Zhang, X., Yang, Z., Bashir, M. & Lee, K. Collision avoidance for autonomous ship using deep reinforcement learning and prior-knowledge-based approximate representation. *Frontiers in Marine Science* **9**, 1084763 (2023).

[13] Zhang, X., Wang, C., Jiang, L., An, L. & Yang, R. Collision-avoidance navigation systems for maritime autonomous surface ships: A state of the art survey. *Ocean Engineering* **235**, 109380 (2021).

[14] Zheng, K., Zhang, X., Wang, C., Zhang, M. & Cui, H. A partially observable multi-ship collision avoidance decision-making model based on deep reinforcement learning. *Ocean & Coastal Management* **242**, 106689 (2023).

[15] Fossen, T. I., Pettersen, K. Y. & Galeazzi, R. Line-of-sight path following for dubins paths with adaptive sideslip compensation of drift forces. *IEEE Transactions on Control Systems Technology* **23**, 820–827 (2014).

[16] Yang, L. *et al.* Autonomous environment-adaptive microrobot swarm navigation enabled by deep learning-based real-time distribution planning. *Nature Machine Intelligence* **4**, 480–493 (2022).

[17] Huang, Y. *et al.* A motion planning and tracking framework for autonomous vehicles based on artificial potential field elaborated resistance network approach. *IEEE Transactions on Industrial Electronics* **67**, 1376–1386 (2019).

[18] Li, Q. *et al.* Metadrive: Composing diverse driving scenarios for generalizable reinforcement learning. *IEEE Transactions on Pattern Analysis and Machine Intelligence* **45**, 3461–3475 (2023).

[19] Feng, S. *et al.* Dense reinforcement learning for safety validation of autonomous vehicles. *Nature* **615**, 620–627 (2023).
